# Supplementary material for: Structure Prediction, Evaluation, and Validation of GPR18 Lipid Receptor Using Free Programs
Source: Int J Mol Sci. 2022 Jul 18;23(14):7917. doi: 10.3390/ijms23147917 (PMC9319093; doi:10.3390/ijms23147917)

International Journal of *Molecular Sciences*

MDPI

Article – Supplementary material

Structure prediction, evaluation, and validation of GPR18 lipid receptor using free programs

Ilona Michalik †, Kamil J. Kuder *,†, Katarzyna Kieć-Kononowicz and Jadwiga Handzlik

1. Protein templates and sequence alignments used for models' generation

1.1. Classical method

Table S1 Templates used in the classical method for homology modelling of the GPR18 receptor to-gether with parameters obtained using BLAST and HHblits searches

		**δ-type opioid receptor**	**κ-type opioid receptor**	**Nociceptin receptor**
Structure	PDB ID	4N6H.A	4DJH.A	5DHH.A
	Method		X-ray	
	Resolution [Å]	1.8	2.9	3.0
	Degree active [%]		6 (inactive state)	
	Ligand - PDB ID, name function	EJ4, naltrindole, antagonist	JDC, JDTic, antagonist	DGW, SB 612111, antagonist
	Sodium in structure	Yes	No	No
BLAST	Global Model Quality Estimate	0.46	0.37	-
	Sequence identity [%]	26.99	27.42	-
	Sequence coverage [%]	68 (range: 23-250)	56 (range: 29-214)	-
	Sequence similarity [%]	36	36	-
HHblits	Global Model Quality Estimate	0.58	0.56	0.57
	Sequence identity [%]	22.70	23.78	23.59
	Sequence coverage [%]	85 (range: 22-304)	86 (range: 19-304)	86 (range: 21-305)
	Sequence similarity [%]	33	33	32

```
Reference sequence (1): Q14330|hGPR18
Identities normalised by aligned length.
Colored by: property

                    cov     pid   1 [         .         .         .         .         :         . 60
1 Q14330|hGPR18 100.0% 100.0%     MITLNNQDQPVPFNSSHPDEYKIAALVFYSCIFIIGLFVNITALWVFSCTTKKRTTVTIY
2 4N6H_A_CB5     89.4%  21.1%     --------GSPGARSASSLALAIAITALYSAVCAVGLLGNVLVMFGIVRYTKMKTATNIY
3 5DHH_A         83.7%  22.8%     ---------------FLPLGLKVTIVGLYLAVCVGGLLGNCLVMYVILRHTKMKTATNIY
4 4DJH_A         85.2%  22.0%     -----------------SPAIPVIITAVYSVVFVVGLVGNSLVMFVIIRYTKMKTATNIY

                    cov     pid  61           .         .         .         1         .         . 120
1 Q14330|hGPR18 100.0% 100.0%     MMNVALVDLIFIMTLPFRMFYYAKDEWPFGEYFCQILGALTVFYPSIALWLLAFISADRY
2 4N6H_A_CB5     89.4%  21.1%     IFNLALADALATSTLPFQSAKYLMETWPFGELLCKAVLSIDYYNMFTSIFTLTMMSVDR-
3 5DHH_A         83.7%  22.8%     IFNLALADTLVLLTLPFQGTDILLGFWPFGNALCKTVIAIDYYNMFTSTFTLTAMSVDRY
4 4DJH_A         85.2%  22.0%     IFNLALADALVTTTMPFQSTVYLMNSWPFGDVLCKIVLSIDYYNMFTSIFTLTMMSVDRY

                    cov     pid 121           .         .         :         .         .         . 180
1 Q14330|hGPR18 100.0% 100.0%     MAIVQPKYAKELKNTCKAVLACVGVWIMTLTTTTPLLLLYKDPDKDSTPATCLKISDIIY
2 4N6H_A_CB5     89.4%  21.1%     YIVCHPVKALDFRTPAKAKLINICIWVLASGVGVPIMVMAVTRPRDGAVVCMLQFPSPS-
3 5DHH_A         83.7%  22.8%     VAICHP------RTSSKAQAVNVAIWALASVVGVPVAIMGSAQVEDEEIECLVEIPTPQD
4 4DJH_A         85.2%  22.0%     IAVCHPVKALDFRTPLKAKIINICIWLLSSSVGISAIVLGGTKVREDVDVIECSLQFPDD

                    cov     pid 181           .         2         .         .         .         . 240
1 Q14330|hGPR18 100.0% 100.0%     --LKAVNVLNLTRLTFFFLIPLFIMIGCYLVIIHNLLHG---RTSKLKPKVKEKSIRIII
2 4N6H_A_CB5     89.4%  21.1%     --WYWDTVTKICVFLFAFVVPILIITVCYGLMLLRLRSVRLLSGSKEKDRSLRRITRMVL
3 5DHH_A         83.7%  22.8%     ---YWGPVFAICIFLFSFIVPVLVISVCYSLMIRRLRGVRLLSGSREKDRNLRRITRLVL
4 4DJH_A         85.2%  22.0%     DYSWWDLFMKICVFIFAFVIPVLIIIVCYTLMILRLKSVRLLSG-REKDRNLRRITRLVL

                    cov     pid 241           :         .         .         .         .       3 300
1 Q14330|hGPR18 100.0% 100.0%     TLLVQVLVCFMPFHICFAFLMLG---TGENSYNPWGAFTTFLMNLSTCLDVILYYIVSKQ
2 4N6H_A_CB5     89.4%  21.1%     VVVGAFVVCWAPIHIFVIVWTLVDIDRRDPLVVAALHLCIALGYANSSLNPVLYAFLDEN
3 5DHH_A         83.7%  22.8%     VVVAVFVGCWTPVQVFVLAQGLG-VQPSSETAVAILRFCTALGYVNSCLNPILYAFLDEN
4 4DJH_A         85.2%  22.0%     VVVAVFVVCWTPIHIFILVEALG------SAALSSYYFCIALGYTNSSLNPILYAFLDEN

                    cov     pid 301           .         .         .      ] 339
1 Q14330|hGPR18 100.0% 100.0%     FQARVISVMLYRNYLRSMRRKSFRSGSLRSLSNINSEML
2 4N6H_A_CB5     89.4%  21.1%     FKRCFRQLCRKPCG-------------------------
3 5DHH_A         83.7%  22.8%     FKACFRK--------------------------------
4 4DJH_A         85.2%  22.0%     FKRCFRDFCFP----------------------------
```

Figure S1 Sequence alignments of selected opioid receptors and GPR18. Top panel: alignment with 1 template generated using Clustal Omega; bottom panel: alignment with 3 templates by the Clustal W algorithm. Visualisation - the GPCR colour map.

1.2 Threading method

Table S2 Protein structure templates used for models' generation with threading method

Receptor name*	PDB ID	Resolution [Å] (method)	Degree active [%]	Ligand – PDB ID
**Neuropeptide Y receptor type 1 (Y_1)**	5ZBH	3.0 (X-ray)	1 (inactive)	9AF (antagonist)
**δ-type opioid receptor**	4N6H	1.8 (X-ray)	6 (inactive)	EJ4 (naltrindole, antagonist)
**κ-type opioid receptor**	4DJH	2.9 (X-ray)	6 (inactive)	JDC (antagonist)
**Apelin receptor**	5VBL	2.6 (X-ray)	7 (inactive)	- (agonist)

**C5a anaphylatoxin chemotactic receptor 1 ($C5aR_1$)**	5O9H	2.7 (X-ray)	9 (inactive)	9P2 (antagonist)
**C-C chemokine receptor type 5 (CCR5)**	4MBS	2.7 (X-ray)	29 (inactive)	MRV (antagonist)
**P2Y purinoceptor 1 ($P2Y_1$)**	4XNV	2.2 (X-ray)	39 (intermediate)	BUR (allosteric antagonist)
**P2Y purinoceptor 1 ($P2Y_1$)**	4XNW	2.7 (X-ray)	39 (intermediate)	2ID (antagonist)
**Proteinase-activated receptor 2 (PAR2)**	5NDD	2.8 (X-ray)	39 (intermediate)	8TZ (antagonist)
**Mouse μ-type opioid receptor**	5C1M	2.1 (X-ray)	94 (active)	VF1 (agonist)
**N-formyl peptide receptor 2 (FPR2/ALX)**	6LW5	2.8 (X-ray)	94 (active)	- (WKYMVm, agonist)
**C-C chemokine receptor type 6 (CCR6)**	6WWZ	3.3 (cryo-EM)	100 (active)	- (CCL20, agonist)
**Substance-P receptor (NK_1)**	2KS9	- (NMR)	-	-
**C-X-C chemokine receptor type 1 (CXCR1)**	2LNL	- (NMR)	-	-

* All receptors, with the exception of opioid μ, are human receptors.

Table S3 Ranking of the templates created by the LOMETS package of the stand-alone version of I-TASSER, which have been taken into account in the further model development process

**Threading program**	**Rank**	**PDB ID**	**Sequence identity [%]**	**Sequence coverage [%]**	**Normalized Z-score ***
MUSTER	1	4N6H.A	21.6	89.4	3.38
dPPAS	2	4N6H.A	21.6	89.4	4.18
wdPPAS	3	2LNL.A	24.4	86.7	4.32
wMUSTER	4	4N6H.A	22.9	88.2	3.93
wPPAS	5	5VBL.B	24.1	86.4	5.39
dPPAS2	6	4N6H.A	21.6	89.4	4.13
PPAS	7	4N6H.A	21.6	89.4	3.58
Env-PPAS	8	4N6H.A	21.6	89.4	4.67

Threading program	Rank	PDB ID	Sequence identity [%]	Sequence coverage [%]	Normalized Z-score *
MUSTER	9	4MBS.A	21.2	87.0	3.31
dPPAS	10	4MBS.A	20.5	87.0	4.12
wdPPAS	11	4MBS.A	21.3	86.7	4.32
wMUSTER	12	4MBS.A	21.7	86.4	3.88
wPPAS	13	6LW5.A	23.6	89.4	5.39
dPPAS2	14	4MBS.A	20.5	87.0	4.07
PPAS	15	6WWZ.F	23.2	90.0	3.53
Env-PPAS	16	6WWZ.F	23.2	90.0	4.47
MUSTER	17	5O9H.A	21.9	87.0	3.25
dPPAS	18	5O9H.A	21.9	87.0	3.96
wdPPAS	19	4XNV.A	26.0	84.9	4.32
wMUSTER	20	6WWZ.F	23.2	89.7	3.83

* A Normalized Z-score >1 means a good alignment.

Table S4 Ranking of the templates created by the LOMETS package in the I-TASSER web service, which were taken into account in the further model development process

Threading program	Rank	PDB ID	Sequence identity [%]	Sequence coverage [%]	Normalized Z-score*
MUSTER	1	4N6H.A	22	89	3.20
FFAS-3D	2	4XNW.A	24	88	4.36
SPARKS-X	3	4N6H.A	22	89	3.55
HHSEARCH2	4	5ZBH.A	19	87	1.12
HHSEARCH I	5	4DJH.A	24	85	0.85
Neff-PPAS	6	4N6H.A	23	89	3.01
HHSEARCH	7	5ZBH.A	20	86	1.24
pGenTHREADER	8	5C1M.A	24	88	5.74
wdPPAS	9	4MBS.A	21	87	2.98
PROSPECT2	10	4N6H.A	22	89	4.06

* A Normalized Z-score >1 means a good alignment.

Table S5 Ranking of the templates created by the LOMETS package in the C-I-TASSER web service, which were taken into account in the further model development process

Threading program	Rank	PDB ID	Sequence identity [%]	Sequence coverage **	Normalized Z-score*
SPARKS-K	1	4N6H.A	22	6.11	3.52
FFAS-3D	2	4XNW.A	24	6.59	3.79
HHpred	3	6WWZ.F	24	6.61	6.09
MUSTER	4	4N6H.A	22	6.11	3.20
CNFpred	5	5C1M.A	26	6.71	2.13
HHsearch-2	6	5ZBH.A	19	5.36	0.60
Neff-PPAS	7	4N6H.A	23	6.35	2.99
HHsearch	8	2KS9.A	15	5.07	0.94
PROSPECTOR2	9	4N6H.A	22	6.11	2.89
SAM	10	5NDD.A	23	6.53	4.67

* A Normalized Z-score >1 means a good alignment.

** Sequence coverage is equal the number of aligned template residues divided by query sequence length.

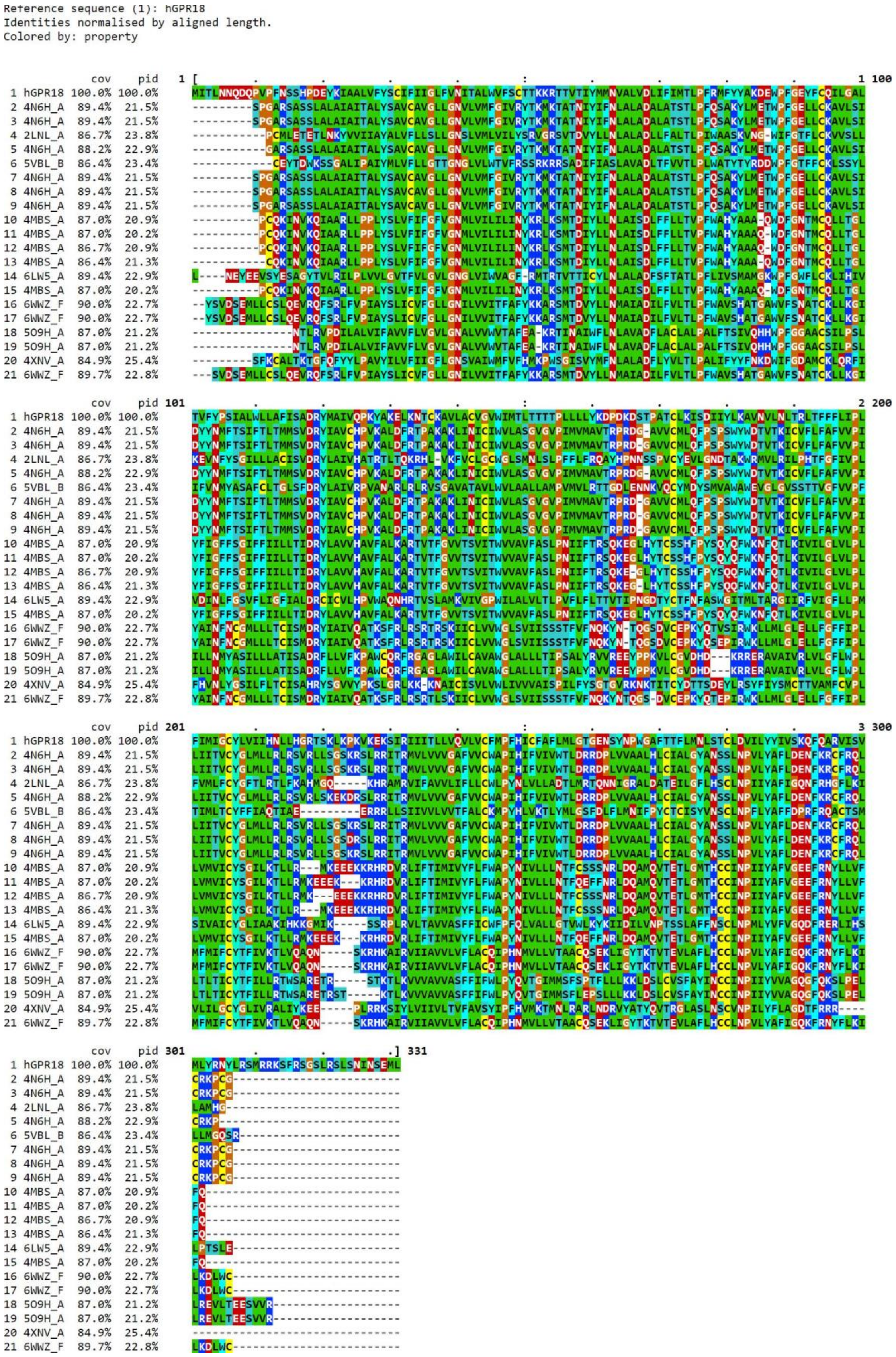

Figure S2 Sequence alignment of selected templates and GPR18 receptor using programs from the LOMETS package (stand-alone version of I-TASSER]). Visualisation - the GPCR colour map.

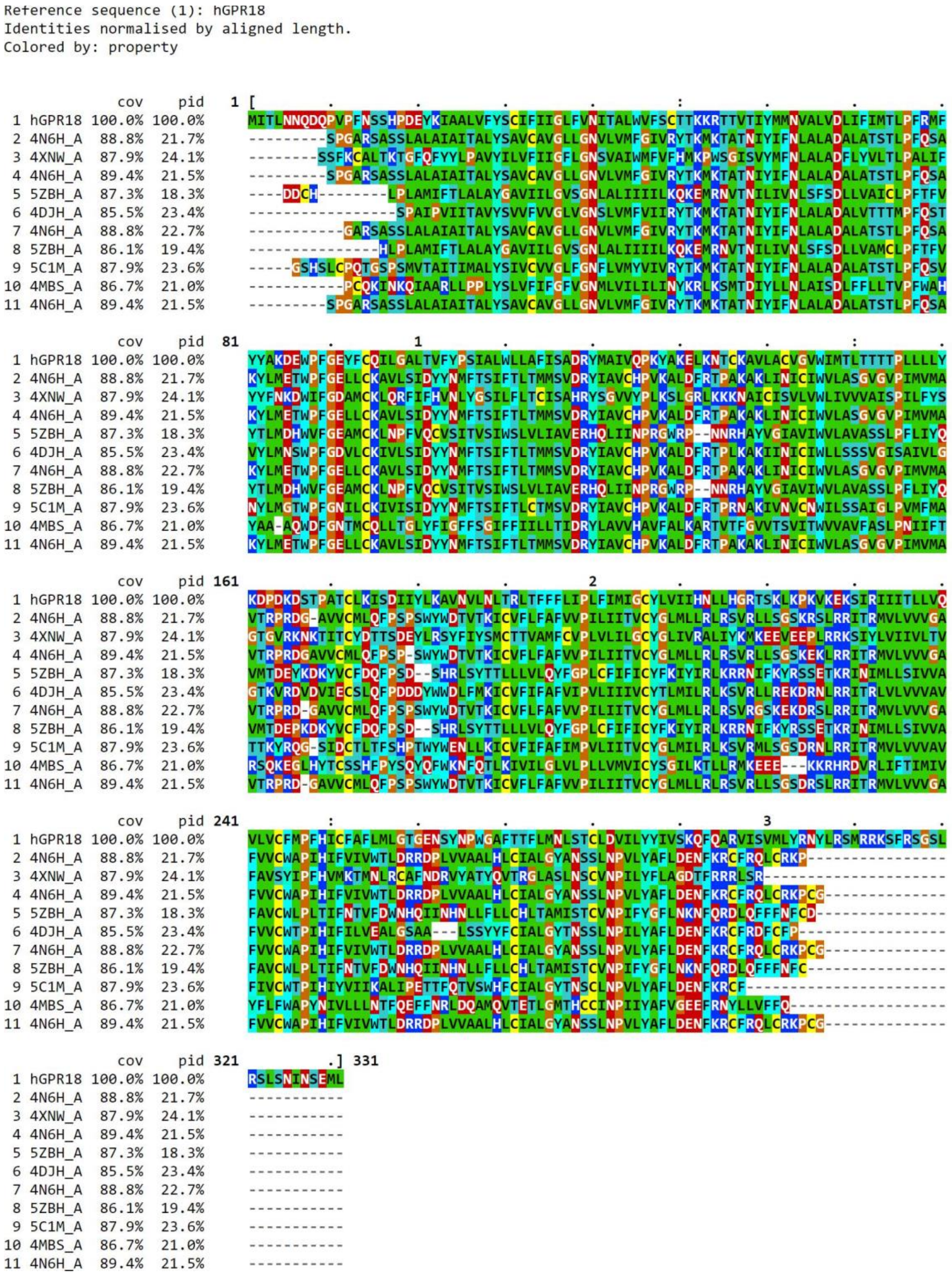

Figure S3 Sequence alignment of selected templates and GPR18 receptor using programs from the LOMETS package (I-TASSER web server). Visualisation - the GPCR colour map.

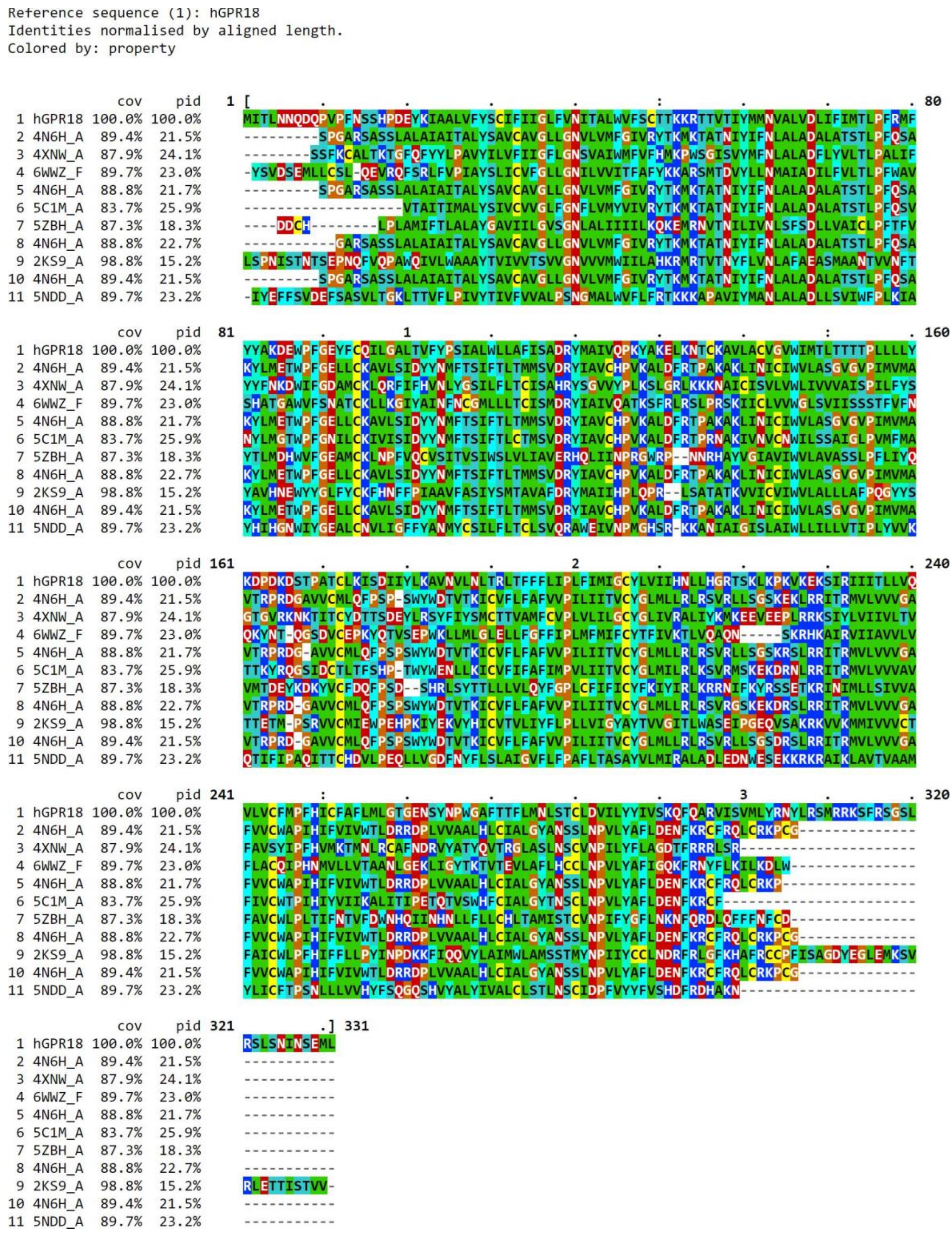

Figure S4 Sequence alignment of selected templates and GPR18 receptor using programs from the LOMETS package (C-I-TASSER web server). Visualisation - the GPCR colour map.

1.3 Ab initio method

Table S6 Templates used in the *ab initio* (trRosetta) method together with the values of the individual parameters calculated by the trRosetta algorithms.

	**Leukotriene B4 receptor 1 (BLT$_1$)**	**Neurotensin receptor type 1 (NTS$_1$)**		**Proteinase-activated receptor 1 (PAR1)**	**C-X-C chemokine receptor type 4 (CXCR4)**
**PDB ID**	5X33.A	5T04.A	4GRV.A	3VW7.A	3ODU.A
**Method**	X-ray				
**Resolution [Å]**	3.7	3.3	2.8	2.2	2.5
**Species**	*Cavia porcellus* (Guinea pig)	*Rattus norvegicus* (Rat)		*Homo sapiens* (Human)	
**Degree active [%]**	7 (inactive)	85 (active)	85 (active)	39 (intermediate)	7 (inactive)
**Ligand - PDB ID**	7Y9 (antagonist)	- (neurotensin, agonist)		VPX (antagonist)	ITD (antagonist)
**Confidence [%]**	100	100	100	100	100
**Sequence identity [%]**	24.2	24.8	25.4	25.1	26.0
**Sequence coverage [%]**	81.9	84.6	84.6	84	90
**E-value**	8.60^{-44}	5.30^{-44}	2.60^{-43}	1.70^{-43}	9.00^{-43}
**Z-score**	10.922	10.912	10.813	10.803	10.688

Confidence - The probability of a template to be a true positive.
Coverage - The number of non-gap alignment divided by the query sequence length.
Identity - The number of aligned identical residues divided by the query sequence length.
E-value - Statistical significance of the alignment. An E-value closer to 0 indicates a more significant hit.
Z-score - Normalized score of the raw alignment score. A Z-score >11 indicates a reliable hit.

```
Reference sequence (1): hGPR18
Identities normalised by aligned length.
Colored by: property


                  cov     pid   1 [        .         .         .         .         :         . 60
1 hGPR18 100.0% 100.0%      MITLNNQDQPVPFNSSHPDEYKIAALVFYSCIFIIGLFVNITALWVFSCTTKKRTTVTIY
2 5X33_A  81.9%  18.7%      ----------------SNTFIPLLAMILLSVSMVVGLPGNTFVVWSILKRMRKRSVTALM
3 5T04_A  84.6%  19.1%      -TSESTAGPNSDLDVNTDIYSKVLVTAIYLALFVVGTVGNSVTLFTLAR-----STVHYH
4 4GRV_A  84.6%  19.9%      ---------NSDLDVNTDIYSKVLVTAIYLALFVVGTVGNSVTLFTLARK----STVHYH
5 3VW7_A  84.0%  21.6%      ----------DASGYLTSSWLTLFVPSVYTGVFVVSLPLNIMAIVVFILKMKVKKPAVVY
6 3ODU_A  90.0%  24.2%      ----------PCFREENANFNKIFLPTIYSIIFLTGIVGNGLVILVMGYQKKLRSMTDKY

                  cov     pid  61          .         .         .         1         .         . 120
1 hGPR18 100.0% 100.0%      MMNVALVDLIFIMTLPFRMFYYAKDEWPFGEYFCQILGALTVFYPSIALWLLAFISADRY
2 5X33_A  81.9%  18.7%      VLNLALADLAVLLTAPFFLHFLTWGTWSFGLAGCRLCHYICGVSMYASVLLITAMSLDRS
3 5T04_A  84.6%  19.1%      LGSLALSDLLILLAMPVELYNFIHHPWAFGDAGCRGYYFLRDACTYATALNVASLSVERY
4 4GRV_A  84.6%  19.9%      LGSLALSDLLILLAMPVELYNFIHHPWAFGDAGCRGYYFLRDACTYATALNVASLSVARY
5 3VW7_A  84.0%  21.6%      MLHLATADVLFVSVLPFKISYYFSGDWQFGSELCRFVTAAFYCNMYASILLMTVISIDRF
6 3ODU_A  90.0%  24.2%      RLHLSVADLLFVITLPFWAVDAV-ANWYFGNFLCKAVHVIYTVNLYSSVWILAFISLDRY

                  cov     pid 121          .         .         :         .         .         . 180
1 hGPR18 100.0% 100.0%      MAIVQPKYAKELKNTCKAVLACVGVWIMTLTTTTPLLLLYKDPDKDSTPATCLKISDIIY
2 5X33_A  81.9%  18.7%      LAVASPFLSQKVRTKTAARWLLVGIWGASFLLATPVLAFRKVVKLTNETDLCLAVYPSD-
3 5T04_A  84.6%  19.1%      LAICHPFKAKTLMSRSRTKKFISAIWLASALLAIPMLFTMGLQNRSAGGLVCTPIVDTA-
4 4GRV_A  84.6%  19.9%      LAICHPFKAKTLMSRSRTKKFISAIWLASALLAIPMLFTMGLQNRSPGGLVCTPIVDTA-
5 3VW7_A  84.0%  21.6%      LAVVYPM-----RTLGRASFTCLAIWALAIAGVVPLLLKEQTIQVPLGITTCHDVLSETL
6 3ODU_A  90.0%  24.2%      LAIVHATNSQRPRKLLAEKVVYVGVWIPALLLTIPDFIFANVSE-ADDRYICDRFYPND-

                  cov     pid 181          .         2         .         .         .         . 240
1 hGPR18 100.0% 100.0%      LKAVNVLNLTRLTFFFLIPLFIMIGCYLVIIHNLLHGRTSKLKPKVKEKSIRIIITLLVQ
2 5X33_A  81.9%  18.7%      -RHKAFHLLFEAFTGFVVPFLIVVASYADISRRLRARGSGSNRFHRRRRTGRLVVIIILA
3 5T04_A  84.6%  19.1%      --TVKVVIQVNTFMSFLFPMLVISILNTVIANKLTVMVNIPGRVQALRHGVLVLRAVVIA
4 4GRV_A  84.6%  19.9%      --TVKVVIQVNTFMSFLFPMLVISILNTVIANKLTVMVNIFGSVQALRHGVLVARAVVIA
5 3VW7_A  84.0%  21.6%      EGYYAYYFSAFSAVFFFVPLIISTVCYVSIIRCLSSSANIYANRSKKSRALFLSAAVFCI
6 3ODU_A  90.0%  24.2%      -LWVVVFQFQHIMVGLILPGIVILSCYCIIISKLSHSGSNISKGHQKRKALKTTVILILA

                  cov     pid 241          :         .         .         .         .         3 300
1 hGPR18 100.0% 100.0%      VLVCFMPFHICFAFLMLGTGENSYNPWGAFTTFLMNLSTCLDVILYYIVSKQFQARVISV
2 5X33_A  81.9%  18.7%      FAAFWLPYHVVDLVEGSRVLKQQLRNARKVCIALAFLSSSVNPLLYACA-----------
3 5T04_A  84.6%  19.1%      FVVCWLPYHVRRLMFCYISDEDFYHYFYMLTNALAYASSAINPILYNLV-----------
4 4GRV_A  84.6%  19.9%      FVVCWLPYHVRRLMFCYISDEDFYHYFYMLTNALAYASSAINPILYNLVSANFRQV----
5 3VW7_A  84.0%  21.6%      FIICFGPTNVLLIAHYSFLSHEAAYFAYLLCVCVSSISCCIDPLIYYYASSEC-------
6 3ODU_A  90.0%  24.2%      FFACWLPYYIGISIDSFILLENTVHKWISITEALAFFHCCLNPILYAFLGAKFKTSAQHA

                  cov     pid 301          .         .         .] 331
1 hGPR18 100.0% 100.0%      MLYRNYLRSMRRKSFRSGSLRSLSNINSEML
2 5X33_A  81.9%  18.7%      -------------------------------
3 5T04_A  84.6%  19.1%      -------------------------------
4 4GRV_A  84.6%  19.9%      -------------------------------
5 3VW7_A  84.0%  21.6%      -------------------------------
6 3ODU_A  90.0%  24.2%      LTSGRPLEVLFQ-------------------
```

Figure S5 Alignment of template sequences with the GPR18 receptor using HHsearch [202] by trRo-setta [130]. Visualization with the”GPCR” colour map.

2. Generated GPR18 models qualitative estimation values

Table S7 Results of preliminary estimation of homology models (classical method): MODELLER/PyMod specific values (DOPE score and Objective Function Value), PrankWeb (Pocket score) and SAVES6.0 (values from other columns). The values were coloured with the spectrum: green (best) → red (worst)

Model	DOPE score	Objective Function Value	Pocket score	ERRAT [%]	VERIFY 3D [%]	PROVE	Ramachandran plot [%]				Labelled residues
							Core	allow	gener	disall	
PY_1	-39334.39	1868.94	43.95	72.47	31.72	8.0	88.5	8.2	2.0	1.3	31
PY_2	-39275.60	1750.88	45.82	66.99	36.25	6.7	91.1	6.2	2.0	0.7	24
PY_3	-39464.59	1765.07	44.45	66.35	27.19	6.4	90.5	7.2	0.3	2.0	27
PY_4	-39449.55	2116.05	42.22	58.54	29.00	5.7	91.8	6.2	1.6	0.3	20
PY_5	-39222.86	1754.02	54.70	65.27	35.05	6.3	91.1	6.6	1.3	1.0	23
PY_6	-40796.91	1687.15	50.60	80.58	31.72	6.5	91.5	5.9	1.3	1.3	20
PY_7	-41117.18	1616.13	42.55	73.16	34.74	6.7	92.1	5.2	2.0	0.7	22
PY_8	-40309.58	1706.75	47.01	74.28	37.76	5.9	92.1	4.9	2.0	1.0	21
PY_9	-40612.92	1907.13	40.43	68.15	34.44	5.1	92.5	5.6	1.0	1.0	12
PY_10	-40562.61	1728.90	40.05	76.92	35.05	6.2	92.1	5.2	1.3	1.3	17
PY3_1	-39788.30	10846.41	58.66	58.03	36.25	7.1	91.1	5.6	1.3	2.0	23
PY3_2	-40436.91	10858.64	39.92	60.33	39.88	7.3	92.5	5.2	1.0	1.3	24
PY3_3	-40465.46	10824.43	45.56	57.19	42.30	7.1	92.1	5.2	2.0	0.7	26
PY3_4	-40694.92	10812.47	42.88	61.84	29.00	5.6	91.5	6.6	0.7	1.3	23
PY3_5	-40625.91	10758.70	44.24	57.84	36.86	6.1	91.8	5.2	1.6	1.3	24
PY3_6	-40414.20	10716.33	51.28	57.23	40.18	6.9	92.1	5.9	1.3	0.7	23
PY3_7	-40177.18	10825.27	45.21	59.54	35.05	7.2	90.8	6.9	1.0	1.3	30
PY3_8	-40447.05	10661.49	39.06	61.97	35.65	7.2	93.1	5.2	0.3	1.3	20
PY3_9	-39968.84	10703.59	49.72	63.49	34.74	7.8	93.4	4.9	1.0	0.7	21
PY3_10	-40066.33	10798.45	51.94	63.37	37.16	5.0	94.1	4.3	0.3	1.3	29
PY3_11	-40864.90	10581.10	43.43	54.79	28.40	6.1	93.4	4.6	1.0	1.0	19
PY3_12	-39924.29	10920.07	40.60	52.81	40.18	6.6	91.1	5.6	2.0	1.3	18
PY3_13	-39583.40	10878.23	58.95	50.00	30.51	8.0	92.8	4.9	1.0	1.3	20
PY3_14	-40278.64	10738.23	50.22	48.22	39.88	5.6	93.4	5.2	1.0	0.3	18
PY3_15	-40877.95	10776.21	42.18	64.92	29.91	6.6	91.8	5.6	1.6	1.0	22
PY3_16	-40678.02	10741.28	47.37	63.04	38.07	6.2	90.8	6.6	1.0	1.6	28
PY3_17	-39957.36	10813.73	52.00	61.39	40.18	6.9	92.1	5.2	1.6	1.0	24
PY3_18	-40717.50	10750.69	50.48	49.84	37.46	5.2	94.1	3.6	1.0	1.3	20
PY3_19	-40570.72	10646.63	45.27	61.69	31.42	5.5	92.8	6.2	1.0	0.0	14
PY3_20	-40284.03	10880.83	34.48	67.99	46.53	6.7	92.8	4.6	1.6	1.0	25
PY3_21	-41407.86	10582.94	39.35	69.38	32.63	5.1	90.8	6.9	1.3	1.0	15
PY3_22	-41844.32	10500.78	49.34	76.14	36.86	5.2	94.1	4.6	0.7	0.7	11
PY3_23	-41350.18	10457.73	41.32	73.16	38.07	4.6	94.4	4.6	0.3	0.7	13
PY3_24	-42015.81	10499.40	36.64	83.22	39.58	6.6	94.1	5.2	0.0	0.7	12
PY3_25	-41513.48	10590.32	52.75	81.40	36.56	5.8	92.1	6.2	1.0	0.7	14
PY3_26	-41551.30	10488.67	57.10	83.28	33.53	5.5	93.8	4.6	0.7	1.0	10
PY3_27	-41447.10	10731.27	43.22	79.67	39.88	5.8	91.5	7.5	1.0	0.0	12
PY3_28	-41583.34	10639.54	41.10	72.06	31.72	6.2	92.5	6.2	1.0	0.3	16
PY3_29	-41472.23	10586.89	40.84	83.33	36.86	5.5	91.1	7.2	1.3	0.3	15
PY3_30	-41810.02	10439.98	39.67	79.73	40.18	4.4	94.8	4.3	0.7	0.3	11

Model	DOPE score	Objective Function Value	Pocket score	ERRAT [%]	VERIFY 3D [%]	PROVE	Ramachandran plot [%]				Labelled residues
							Core	allow	gener	disall	
PY3_31	-41727.97	10555.75	38.20	76.90	42.30	4.7	91.8	6.2	1.6	0.3	16
PY3_32	-42348.60	10560.26	40.46	77.08	33.84	5.5	92.1	5.9	1.3	0.7	12
PY3_33	-41799.72	10444.87	47.57	70.79	30.82	4.3	92.1	6.9	0.3	0.7	9
PY3_34	-41444.36	10571.01	37.84	80.00	33.53	6.5	92.5	6.2	1.0	0.3	16
PY3_35	-41625.06	10520.93	54.46	83.88	39.58	5.9	92.5	5.9	0.7	1.0	11
PY3_36	-41445.21	10717.79	49.82	72.37	32.63	6.3	90.8	6.9	0.3	2.0	20
PY3_37	-41736.63	10604.30	46.82	82.72	36.56	3.7	91.8	7.2	0.7	0.3	14
PY3_38	-41626.11	10550.44	53.50	77.89	35.65	4.2	94.1	3.9	1.0	1.0	13
PY3_39	-41751.73	10463.94	41.67	77.05	31.42	5.2	93.8	5.9	0.3	0.0	12
PY3_40	-41879.50	10602.90	48.56	69.21	29.00	5.1	92.5	5.9	0.3	1.3	19

Table S8 Results of the initial estimation of the models that were generated using the threading method: values specific to the I-TASSER/C-I-TASSER program (TM-score and C-score), PrankWeb (Pocket score) and SAVES6.0 (values from the other columns). The values were coloured with the spectrum: green (best) ➞ red (worst).

Model	TM-score*	C-score	Pocket score	ERRAT [%]	VERIFY 3D[%]	PROVE	Ramachandran plot [%]				Labelled residues
							core	allow	gener	disall	
CIT_1	0.87 ±0.07	1.11	57.25	98.45	45.62	5.2	85.6	10.5	1.3	2.6	33
CIT_2		-2.53	36.37	94.12	37.76	5.6	79.7	17.0	2.3	1.0	40
CIT_3		-2.78	44.88	95.83	40.18	7.1	85.6	11.8	2.3	0.3	29
CIT_4		-2.78	57.73	92.88	37.16	4.5	80.3	14.4	2.0	3.3	40
CIT_5		-4.65	56.21	94.72	34.44	7.4	81.6	13.1	2.6	2.6	41
IT_1	0.70 ±0.12	-0.09	52.30	90.63	54.98	5.8	91.8	5.2	1.3	1.6	14
IT_2		-1.21	41.85	94.41	54.68	5.9	92.1	5.2	1.3	1.3	11
IT_3		-2.57	66.85	89.41	50.15	5.8	93.8	4.9	0.7	0.7	11
IT_4		-2.82	45.56	79.11	47.73	7.6	83.3	11.5	2.0	3.3	32
IT_5		-3.30	58.43	82.30	37.46	7.0	87.5	8.2	2.0	2.3	24
IT_6	0.65 ±0.13	-0.52	44.78	90.09	54.38	5.0	80.3	17.0	2.0	0.7	59
IT_7		-1.26	61.12	92.57	55.29	5.3	79.3	15.1	3.6	2.0	47
IT_8		-0.78	47.17	95.98	58.31	4.4	81.0	15.4	3.3	0.3	45
IT_9		-3.28	52.79	95.67	48.34	4.4	79.3	16.7	1.6	2.3	54
IT_10		-2.58	50.47	96.90	48.34	4.8	78.4	16.7	2.6	2.3	55

* TM-score values can only be calculated (at a relatively high level of reliability) for the best model from each cluster of a given prediction.

Table S9 Results of initial model estimation that were generated using the ab initio method: algorithm-specific values (TM-score and plDDT), PrankWeb (Pocket score) and SAVES6.0 (values from other columns). The values were coloured with the spectrum: green (best) ➞ red (worst).

Model	TM-score*	plDDT	Pocket score	ERRAT [%]	VERIFY 3D [%]	PROVE	Ramachandran plot [%]				Labelled residues
							Core	allow	gener	disall	
DM		85.51	0.87	98.12	47.13	2.8	93.1	6.9	0.0	0.0	5
CF-DM		82.18	23.58	86.76	49.24	4.7	90.2	9.8	0.0	0.0	13
PF-DM		76.93	36.09	94.39	41.39	5.3	91.8	7.9	0.0	0.3	15
TR_1	0.70		63.84	99.38	58.61	4.5	95.4	4.6	0.0	0.0	0
TR_2			60.19	98.76	55.89	3.7	94.4	5.6	0.0	0.0	4
TR_3			42.45	98.14	49.55	3.6	95.4	4.6	0.0	0.0	2
TR_4			56.62	97.83	58.01	4.2	94.1	5.9	0.0	0.0	7
TR_5			37.28	94.43	54.68	4.5	95.1	4.9	0.0	0.0	4
TTA_1		74.13	69.70	99.69	73.41	2.8	94.8	4.3	0.7	0.3	4
TTA_2			66.89	97.51	61.93	2.9	92.8	5.9	0.7	0.7	5
TTA_3			42.49	97.50	58.31	3.5	90.8	7.9	1.0	0.3	12
TTA_4			62.57	97.21	71.30	4.0	93.8	4.9	1.0	0.3	7
TTA_5			84.11	98.14	61.33	3.4	94.4	3.9	0.7	1.0	9

* Measure values, such as TM-score and plDDT, can only be calculated (at a relatively high level of reliability) for the best model from each cluster of a given prediction.

3. All models ranking

Table S10 Numerical estimates of all models including values from SAVES, 3DRefine, DeepRefiner and AUC values from enrichment tests. In each case, the N and C-terminus was shortened (to the sequence range: 11-304) for more objective evaluation. The values were coloured with the spectrum: green (best) ➞ red (worst).

MODEL	ERRAT [%]	VER-IFY 3D [%]	PROVE [%]	Ramach. plot (*core, disall* [%]; *labell. residues*)			RW+ [kcal/mol]	Mol Prob.	Rosetta energy scores	DFIRE scores	GOAP scores	OPUS-PSP scores	Pred. global quality
AF-DM -	99,30	53,06	2,9	95,2	0,0	0	-79353	0,650		-656,4			
CF-DM -	88,38	52,04	4,6	94,1	0,0	10	-76913	1,550	-704,3	-634,9	-38594	-5771	0,199
PF-DM -	95,71	44,56	5,4	93,0	0,4	11	-76733	1,460	-712,9	-633,5	-36489	-5661	0,184
CF-DM 1	100,00	63,61	3,4	92,6	0,0	1	-79184	1,030	-747,4	-650,6	-38838	-5863	0,224
CF-DM 2	100,00	69,73	3,0	94,8	0,0	1	-80041	1,030	-744,7	-657,8	-38909	-5917	0,`219
CF-DM 3	100,00	67,01	2,8	94,1	0,0	2	-79437	0,976	-772,5	-653,4	-38826	-5905	0,218
CF-DM 4	99,65	62,24	3,5	93,7	0,0	0	-79508	0,841	-763,6	-653,4	-38904	-6025	0,218
CF-DM 5	100,00	62,24	2,8	93,0	0,0	3	-79359	0,913	-745,8	-652,5	-38960	-5834	0,218
AF-DM 1	99,65	65,31	3,6	95,9	0,0	1	-80278	0,913	-799,0	-659,2	-37888	-6148	0,212
AF-DM 2	100,00	62,93	3,2	96,3	0,0	2	-80153	0,880	-805,0	-657,6	-37483	-6160	0,209
AF-DM 3	100,00	68,03	4,3	95,6	0,0	4	-80548	0,946	-817,3	-660,8	-37194	-6137	0,207
AF-DM 4	100,00	57,14	3,3	94,8	0,0	2	-80677	0,801	-810,1	-662,0	-37980	-6214	0,206
AF-DM 5	100,00	58,50	2,8	95,9	0,0	3	-80314	0,913	-802,8	-659,4	-37885	-6080	0,204
PF-DM 1	100,00	70,07	4,3	93,0	0,0	3	-80245	0,976	-760,8	-659,8	-37867	-5791	0,199
PF-DM 2	100,00	61,56	4,4	93,3	0,0	1	-79385	1,077	-732,5	-652,1	-38084	-5882	0,196
PF-DM 3	100,00	59,52	4,6	92,6	0,0	5	-80514	1,077	-766,9	-661,8	-36911	-5752	0,192
PF-DM 4	99,65	68,03	4,5	93,3	0,0	2	-79332	1,077	-754,1	-651,6	-37728	-5810	0,190
PF-DM 5	100,00	65,65	4,5	92,6	0,0	4	-79597	1,003	-760,9	-652,9	-37273	-5862	0,190
TR_1 1	100,00	68,71	2,4	96,3	0,0	2	-79066	1,030	-751,3	-646,8	-37045	-5874	0,263
TR_1 2	99,65	62,59	2,4	96,3	0,0	1	-78938	0,913	-750,0	-646,3	-36575	-5886	0,256

MODEL		ERRAT [%]	VER-IFY 3D [%]	PROVE [%]	Ramach. plot (*core, disall* [%]; *labell. residues*)			RW+ [kcal/ mol]	Mol Prob.	Rosetta energy scores	DFIRE scores	GOAP scores	OPUS-PSP scores	Pred. global quality
TR_1	3	100,00	69,05	2,8	95,6	0,0	0	-79057	1,030	-753,8	-647,0	-36331	-5824	0,255
TR_1	4	100,00	59,86	3,1	95,6	0,0	3	-79076	0,976	-755,1	-647,5	-37213	-5858	0,255
TR_1	5	100,00	70,75	2,9	95,6	0,0	1	-79871	0,880	-768,2	-654,3	-37130	-5913	0,255
TR_2	1	99,30	68,71	4,1	95,6	0,0	2	-78629	1,030	-754,0	-643,5	-36150	-5780	0,264
TR_2	2	100,00	70,41	3,2	94,1	0,0	4	-79623	1,077	-760,3	-653,8	-37225	-5807	0,260
TR_2	3	99,65	76,53	2,9	95,2	0,0	1	-78667	1,030	-746,1	-644,7	-36626	-5791	0,259
TR_2	4	99,65	67,01	3,5	95,9	0,0	4	-78851	0,946	-744,2	-646,6	-36319	-5789	0,259
TR_2	5	99,65	72,45	2,6	95,2	0,0	3	-79220	1,077	-752,6	-649,2	-36788	-5795	0,259
TTA_1	1	100,00	86,73	2,9	92,6	1,1	6	-79115	1,043	-737,1	-649,6	-37530	-5775	0,253
TTA_1	2	99,65	78,23	2,8	94,8	1,1	5	-79477	0,976	-736,7	-652,3	-37702	-5778	0,253
TTA_1	3	100,00	77,89	3,0	93,0	1,1	5	-79231	0,946	-742,0	-650,3	-38216	-5840	0,249
TTA_1	4	99,65	76,87	2,9	93,3	1,1	5	-78693	1,003	-734,7	-645,5	-37026	-5769	0,246
TTA_1	5	100,00	83,33	3,4	94,1	1,1	7	-78979	1,141	-730,2	-648,2	-37748	-5770	0,242
TTA_2	1	100,00	73,13	2,8	94,4	0,4	7	-78996	1,054	-912,9	-650,5	-37851	-5710	0,261
TTA_2	2	100,00	70,75	3,1	94,4	0,4	6	-78888	1,043	-912,1	-649,6	-37885	-5744	0,259
TTA_2	3	100,00	75,85	3,9	94,8	0,7	5	-79063	1,003	-919,8	-651,1	-37940	-5647	0,256
TTA_2	4	100,00	75,51	3,0	94,4	0,4	6	-78902	1,030	-915,2	-649,8	-37968	-5705	0,254
TTA_2	5	99,30	85,03	2,8	94,4	0,4	5	-79023	1,137	-911,2	-650,9	-38115	-5712	0,253
TTA_3	1	99,65	73,81	3,6	92,6	0,4	8	-79268	1,223	-707,9	-651,7	-37220	-5762	0,259
TTA_3	2	100,00	65,31	4,7	91,9	0,0	5	-79178	1,091	-698,7	-649,8	-36261	-5757	0,254
TTA_3	3	100,00	77,21	3,2	93,3	0,0	7	-79049	1,237	-711,8	-649,3	-36820	-5738	0,253
TTA_3	4	99,65	71,77	3,6	94,1	0,0	7	-79085	1,060	-709,1	-649,6	-36489	-5726	0,248
TTA_3	5	97,55	70,07	3,2	93,0	0,4	7	-78970	1,204	-709,5	-648,3	-36817	-5744	0,247
TTA_4	1	99,65	66,67	3,6	94,1	0,4	5	-78102	1,003	-715,5	-640,5	-37024	-5663	0,259
TTA_4	2	99,65	68,37	2,7	94,1	0,4	5	-78849	1,003	-719,3	-646,8	-36932	-5696	0,246
TTA_4	3	100,00	75,85	2,4	93,7	0,4	5	-78195	0,841	-713,1	-640,4	-36797	-5655	0,243
TTA_4	4	100,00	73,13	3,8	92,2	0,4	10	-78622	1,282	-705,8	-645,0	-37866	-5677	0,243
TTA_4	5	100,00	73,47	3,8	93,3	0,4	6	-78681	1,003	-708,2	-645,8	-37641	-5666	0,240
TTA_5	1	98,25	60,88	2,8	93,7	1,1	10	-77692	1,223	-829,4	-639,2	-36053	-5426	0,270
TTA_5	2	98,25	61,90	3,7	93,7	1,1	10	-77692	1,216	-820,7	-636,5	-36265	-5475	0,270
TTA_5	3	98,25	64,97	3,1	93,3	0,7	11	-77644	1,261	-826,3	-638,7	-35917	-5468	0,268
TTA_5	4	98,25	59,52	3,3	93,3	0,7	11	-77574	1,192	-824,7	-638,0	-36033	-5444	0,268
TTA_5	5	98,25	57,14	3,6	94,1	0,7	9	-77516	1,242	-821,6	-637,1	-35924	-5450	0,267
CIT_1	1	100,00	70,07	4,2	93,0	0,4	3	-78343	1,223	-675,5	-642,7	-34523	-5662	0,249
CIT_1	2	100,00	65,65	3,2	94,1	0,4	6	-78045	1,067	-649,7	-639,9	-34767	-5708	0,239
CIT_1	3	100,00	61,22	3,1	93,0	0,4	2	-77786	1,380	-656,4	-637,4	-34974	-5674	0,238
CIT_1	4	100,00	70,41	3,1	92,6	0,4	2	-78293	1,297	-670,2	-642,0	-34866	-5876	0,237
CIT_1	5	98,95	68,71	3,0	93,7	0,4	2	-77574	1,029	-666,6	-636,1	-35305	-5596	0,236
IT_1	1	100,00	70,07	3,5	93,3	1,1%	7	-77960	1,441	-565,3	-638,6	-34363	-5680	0,232
IT_1	2	98,95	55,10	3,3	93,7	1,1	5	-77724	1,521	-589,9	-637,2	-34735	-5638	0,219
IT_1	3	99,30	64,29	3,0	94,1	1,1	7	-78023	1,512	-564,4	-639,7	-34801	-5659	0,214
IT_1	4	100,00	60,20	3,1	93,3	1,1	5	-77704	1,449	-589,7	-637,5	-34339	-5678	0,214
IT_1	5	99,30	54,08	3,4	93,7	1,1	7	-77414	1,497	-571,7	-633,7	-34847	-5554	0,213
IT_2	1	100,00	59,52	3,1	94,4	0,7	6	-77263	1,261	-615,4	-632,2	-34811	-5444	0,230
IT_2	2	98,25	57,82	2,9	94,8	0,7	6	-78145	1,177	-611,8	-639,7	-35212	-5631	0,224
IT_2	3	98,95	62,59	2,9	94,4	0,7	6	-77672	1,342	-595,2	-635,2	-34809	-5636	0,219

MODEL		ERRAT [%]	VER-IFY 3D [%]	PROVE [%]	Ramach. plot (*core, disall* [%]; *labell. residues*)			RW+ [kcal/ mol]	Mol Prob.	Rosetta energy scores	DFIRE scores	GOAP scores	OPUS-PSP scores	Pred. global quality
IT_2	4	100,00	63,27	2,9	94,1	0,7	4	-77595	1,350	-622,5	-634,9	-34782	-5548	0,217
IT_2	5	100,00	66,67	3,1	94,4	0,7	4	-78418	1,067	-610,5	-642,0	-35127	-5673	0,216
IT_3	1	97,20	58,84	3,3	92,2	0,7	6	-77714	1,316	-585,2	-635,4	-34394	-5557	0,241
IT_3	2	97,20	56,80	2,7	94,1	0,7	6	-78058	1,449	-586,2	-638,0	-33441	-5698	0,236
IT_3	3	96,50	70,41	3,1	93,3	0,7	8	-77969	1,449	-590,4	-638,8	-35068	-5670	0,230
IT_3	4	93,68	51,70	3,2	94,4	0,7	6	-77239	1,433	-556,1	-631,6	-34206	-5395	0,228
IT_3	5	95,10	53,40	2,9	93,0	0,7	5	-77224	1,433	-561,8	-631,8	-34680	-5479	0,226
PY_9	1	98,59	75,85	2,9	90,4	0,4	5	-76527	1,366	-606,6	-626,2	-34897	-5568	0,219
PY_9	2	96,47	74,15	3,0	90,7	0,4	6	-76590	1,479	-598,8	-627,8	-34721	-5384	0,214
PY_9	3	98,95	76,53	2,7	91,1	0,4	3	-77011	1,497	-567,1	-631,6	-34164	-5591	0,213
PY_9	4	98,94	74,15	2,9	91,1	0,4	5	-76589	1,553	-573,4	-627,0	-34446	-5458	0,207
PY_9	5	96,50	67,35	3,5	90,0	0,4	4	-76921	1,527	-584,9	-630,3	-34683	-5476	0,204
PY3_10	1	98,60	66,33	3,6	90,7	1,5	11	-76143	1,494	-595,3	-624,5	-34667	-5459	0,222
PY3_10	2	99,30	65,65	4,1	90,4	1,1	10	-75595	1,410	-609,5	-619,3	-35669	-5472	0,219
PY3_10	3	97,90	61,22	3,8	90,7	1,9	10	-76647	1,453	-593,9	-628,6	-34084	-5552	0,218
PY3_10	4	98,95	67,01	3,0	90,4	1,9	10	-76024	1,618	-606,3	-623,4	-33872	-5351	0,218
PY3_10	5	94,41	64,63	3,7	90,4	1,5	9	-76398	1,610	-601,7	-627,1	-34646	-5434	0,217
PY3_30	1	96,85	58,16	4,0	93,0	0,7	6	-76878	1,311	-630,2	-631,1	-34749	-5496	0,225
PY3_30	2	96,49	60,88	3,8	94,1	0,4	5	-76278	1,241	-630,0	-626,3	-35114	-5448	0,225
PY3_30	3	97,19	53,06	2,9	93,3	0,4	4	-76848	1,182	-643,5	-630,7	-34305	-5627	0,225
PY3_30	4	98,94	54,08	3,6	93,7	0,7	4	-75966	1,153	-626,8	-622,4	-34084	-5514	0,223
PY3_30	5	100,00	59,18	4,1	91,9	0,7	4	-76397	1,223	-636,2	-627,5	-34149	-5459	0,222

Table S11 Stars value ranking of all models selected for further optimization (including the DM mod-els)

MODEL		ERRAT	VER-IFY 3D	PROVE	Core, disall*	Labell. resid.	RW+	Mol Prob.	Rosetta Energy scores	DFIRE scores	GOAP scores	OPUS-PSP scores	Pred. global quality
AF-DM	-	★★	-	★★	★★★	★★★	★★★	★★★	-	★★★	-	-	-
CF-DM	-	-	-	-	★★☆	-	-	-	★★	★	★★★	★★★	-
PF-DM	-	-	-	-	★☆	-	-	-	★★	★	★★	★★	-
CF-DM	1	★★★	★	★	★☆	★★★	★★★	★★	★★	★★★	★★★	★★★	★
CF-DM	2	★★★	★★	★★	★★★	★★★	★★★	★★	★★	★★★	★★★	★★★	-
CF-DM	3	★★★	★★	★★★	★★☆	★★★	★★★	★★★	★★★	★★★	★★★	★★★	-
CF-DM	4	★★	★	★	★★☆	★★★	★★★	★★★	★★★	★★★	★★★	★★★	-
CF-DM	5	★★★	★	★★★	★★	★★★	★★★	★★★	★★	★★★	★★★	★★★	-
AF-DM	1	★★	★	★	★★★	★★★	★★★	★★★	★★★	★★★	★★★	★★★	-
AF-DM	2	★★★	★	★★	★★★	★★★	★★★	★★★	★★★	★★★	★★★	★★★	-
AF-DM	3	★★★	★★	-	★★★	★★	★★★	★★★	★★★	★★★	★★	★★★	-

MODEL		ERRAT	VER-IFY 3D	PROVE	Core, disall*	Labell. resid.	RW+	Mol Prob.	Rosetta Energy scores	DFIRE scores	GOAP scores	OPUS-PSP scores	Pred. global quality
AF-DM	4	★★★	-	★	★★★	★★★	★★★	★★★	★★★	★★★	★★★	★★★	-
AF-DM	5	★★★	-	★★★	★★★	★★★	★★★	★★★	★★★	★★★	★★★	★★★	-
PF-DM	1	★★★	★★	-	★★	★★★	★★★	★★★	★★★	★★★	★★★	★★★	-
PF-DM	2	★★★	★	-	★★	★★★	★★★	★★	★★	★★★	★★★	★★★	-
PF-DM	3	★★★	-	-	★☆	★★	★★★	★★	★★★	★★★	★★	★★	-
PF-DM	4	★★	★★	-	★★	★★★	★★★	★★	★★★	★★★	★★★	★★★	-
PF-DM	5	★★★	★	-	★☆	★★	★★★	★★	★★★	★★★	★★★	★★★	-
TR_1	1	★★★	★★	★★★	★★★	★★★	★★★	★★	★★★	★★	★★	★★★	★★★
TR_1	2	★★	★	★★★	★★★	★★★	★★	★★★	★★★	★★	★★	★★★	★★
TR_1	3	★★★	★★	★★★	★★★	★★★	★★★	★★	★★★	★★	★★	★★★	★★
TR_1	4	★★★	-	★★	★★★	★★★	★★★	★★★	★★★	★★	★★★	★★★	★★
TR_1	5	★★★	★★	★★	★★★	★★★	★★★	★★★	★★★	★★★	★★	★★★	★★
TR_2	1	★★	★★	-	★★★	★★★	★★	★★	★★★	★★	★★	★★★	★★★
TR_2	2	★★★	★★	★★	★★☆	★★	★★★	★★	★★★	★★★	★★★	★★★	★★★
TR_2	3	★★	★★★	★★	★★★	★★★	★★	★★	★★	★★	★★	★★★	★★
TR_2	4	★★	★★	★	★★★	★★	★★	★★★	★★	★★	★★	★★★	★★
TR_2	5	★★	★★	★★★	★★★	★★★	★★★	★★	★★★	★★★	★★	★★★	★★
TTA_1	1	★★★	★★★	★★	-	★	★★★	★★	★★	★★★	★★★	★★★	★★
TTA_1	2	★★	★★★	★★★	★☆	★★	★★★	★★★	★★	★★★	★★★	★★★	★★
TTA_1	3	★★★	★★★	★★	☆	★★	★★★	★★★	★★	★★★	★★★	★★★	★★
TTA_1	4	★★	★★★	★★	☆	★★	★★	★★	★★	★★	★★	★★	★★
TTA_1	5	★★★	★★★	★	★	★	★★	★★	★★	★★	★★★	★★	★★
TTA_2	1	★★★	★★★	★★★	★★☆	★	★★	★★	★★★	★★★	★★★	★★	★★★
TTA_2	2	★★★	★★	★★	★★☆	★	★★	★★	★★★	★★★	★★★	★★	★★
TTA_2	3	★★★	★★★	-	★★	★★	★★★	★★	★★★	★★★	★★★	★	★★
TTA_2	4	★★★	★★★	★★	★★☆	★	★★	★★	★★★	★★★	★★★	★★	★★
TTA_2	5	★★	★★★	★★★	★★☆	★★	★★★	★★	★★★	★★★	★★★	★★	★★
TTA_3	1	★★	★★★	★	★	-	★★★	★	★★	★★★	★★★	★★	★★
TTA_3	2	★★★	★	-	★☆	★★	★★★	★★	★★	★★★	★★	★★	★★
TTA_3	3	★★★	★★★	★★	★★	★	★★★	★	★★	★★★	★★	★★	★★
TTA_3	4	★★	★★	★	★★☆	★	★★★	★★	★★	★★★	★★	★★	★★

MODEL	ERRAT	VER-IFY 3D	PROVE	Core, disall*	Labell. resid.	RW+	Mol Prob.	Rosetta Energy scores	DFIRE scores	GOAP scores	OPUS-PSP scores	Pred. global quality
TTA_3 5	-	★★	★★	★✩	★	★★	★	★★	★★	★★	★★	★★
TTA_4 1	★★	★	★	★★	★★	★	★★	★★	★	★★	★★	★★
TTA_4 2	★★	★★	★★★	★★	★★	★★	★★	★★	★★	★★	★★	★★
TTA_4 3	★★★	★★★	★★★	★★	★★	★	★★★	★★	★	★★	★★	★★
TTA_4 4	★★★	★★★	-	★	-	★★	★	★★	★★	★★★	★★	★★
TTA_4 5	★★★	★★★	-	★✩	★	★★	★★	★★	★★	★★★	★★	★★
TTA_5 1	★	-	★★★	★	-	★	★	★★★	★	★★	-	★★★
TTA_5 2	★	★	-	★	-	★	★	★★★	★	★★	-	★★★
TTA_5 3	★	★	★★	★	-	★	★	★★★	★	★	-	★★★
TTA_5 4	★	-	★	★	-	★	★★	★★★	★	★★	-	★★★
TTA_5 5	★	-	★	★✩	-	★	★	★★★	★	★	-	★★★
CIT_1 1	★★★	★★	-	★✩	★★★	★★	★	★★	★★	-	★★	★★
CIT_1 2	★★★	★	★★	★★	★	★	★★	★	★	-	★★	★
CIT_1 3	★★★	★	★★	★✩	★★★	★	★	★	★	★	★★	★
CIT_1 4	★★★	★★	★★	★	★★★	★★	★	★	★★	★	★★★	★
CIT_1 5	★	★★	★★	★★	★★★	★	★★	★	★	★	★	★
IT_1 1	★★★	★★	★	✩	★	★	-	-	★	-	★★	★
IT_1 2	★	-	★	★	★★	★	-	-	★	-	★	-
IT_1 3	★★	★	★★	★	★	★	-	-	★	★	★★	-
IT_1 4	★★★	-	★★	✩	★★	★	-	-	★	-	★★	-
IT_1 5	★★	-	★	★	★	-	-	-	★	★	★	-
IT_2 1	★★★	-	★★	★★	★	-	★	★	-	★	-	★
IT_2 2	★	-	★★	★★	★	★	★★	★	★	★	★	★
IT_2 3	★	★	★★	★★	★	★	★	-	★	★	★	-
IT_2 4	★★★	★	★★	★✩	★★	★	★	★	★	-	★	-
IT_2 5	★★★	★	★★	★★	★★	★★	★★	★	★★	★	★★	-
IT_3 1	-	-	★	✩	★	★	★	-	★	-	★	★★
IT_3 2	-	-	★★★	★✩	★	★	-	-	★	-	★★	★
IT_3 3	-	★★	★★	★	-	★	-	-	★	★	★★	★
IT_3 4	-	-	★★	★★	★	-	-	-	-	-	-	★
IT_3 5	-	-	★★	★	★★	-	-	-	-	-	-	★

MODEL	ERRAT	VER-IFY 3D	PROVE	Core, disall*	Labell. resid.	RW+	Mol Prob.	Rosetta Energy scores	DFIRE scores	GOAP scores	OPUS-PSP scores	Pred. global quality
PY_9 1	★	★★★	★★	★	★★	-	★	★	-	★	★	-
PY_9 2	-	★★★	★★	★	★	-	-	-	-	-	-	-
PY_9 3	★	★★★	★★★	★	★★★	-	-	-	-	-	★	-
PY_9 4	★	★★★	★★	★	★★	-	-	-	-	-	-	-
PY_9 5	-	★★	★	★	★★	-	-	-	-	-	-	-
PY3_10 1	★	★	★	-	-	-	-	-	-	-	-	★
PY3_10 2	★★	★	-	-	-	-	-	★	-	★	-	-
PY3_10 3	-	★	-	-	-	-	-	-	-	-	★	-
PY3_10 4	★	★★	★★	-	-	-	-	★	-	-	-	-
PY3_10 5	-	★	-	-	-	-	-	★	-	-	-	-
PY3_30 1	-	-	-	★	★	-	★	★	-	-	-	★
PY3_30 2	-	-	-	★★	★★	-	★	★	-	★	-	★
PY3_30 3	-	-	★★	★⋆	★★	-	★★	★	-	-	★	★
PY3_30 4	★	-	★	★⋆	★★	-	★★	★	-	-	-	★
PY3_30 5	★★★	-	-	⋆	★★	-	★	★	-	-	-	★

*the small star symbol indicates a half value

Table S12 Summary of stars ranking system of all models selected for further optimization (including the DM models)

Model	Total stars	V1	Distance between R119 & S230 [Å][1]	V2	V1+V2	AUC	V3	V1+V2+V3
CIT_1 4	22,0	★★	2,09	★★★	5	0,811	★★★	8
CF-DM 5	29,0	★★★	3,35	★★	5	0,794	★★★	8
PF-DM 1	28,0	★★★	2,16	★★★	6	0,783	★★	8
TTA_5 1	16,0	★	2,97	★★	3	0,808	★★★	6
IT_1 4	11,5	-	2,41	★★★	3	0,797	★★★	6
PY3_10 1	4,0	-	1,94	★★★	3	0,791	★★★	6
PF-DM 4	26,0	★★	3,49	★★	4	0,787	★★	6
PF-DM 5	24,5	★★	2,24	★★★	5	0,767	★	6
TR_1 5	32,0	★★★	2,95	★★	5	0,763	★	6
AF-DM 3	27,0	★★★	2,18	★★★	6	0,564	-	6
AF-DM 2	30,0	★★★	1,93	★★★	6	0,555	-	6
AF-DM 4	28,0	★★★	2,03	★★★	6	0,446	-	6
AF-DM 5	30,0	★★★	2,01	★★★	6	0,394	-	6
CF-DM -	11,5	-	3,40	★★	2	0,834	★★★	5

Model	Total stars	V1	Distance between R119 & S230 [Å][1]	V2	V1+V2	AUC	V3	V1+V2+V3
TTA_1 1	27,0	★★★	4,97	-	3	0,783	★★	5
TTA_4 2	25,0	★★	4,03	★	3	0,781	★★	5
TTA_2 1	30,5	★★★	5,06	-	3	0,777	★★	5
TR_2 5	31,0	★★★	5,04	-	3	0,773	★★	5
TR_2 3	28,0	★★★	4,46	★	4	0,761	★	5
CF-DM 4	27,5	★★★	3,17	★★	5	0,737	-	5
CF-DM 2	29,0	★★★	3,04	★★	5	0,724	-	5
CF-DM 3	31,5	★★★	3,45	★★	5	0,723	-	5
PF-DM 3	21,5	★★	2,29	★★★	5	0,716	-	5
CF-DM 1	26,5	★★	2,39	★★★	5	0,659	-	5
PF-DM 2	25,0	★★	2,23	★★★	5	0,650	-	5
PF-DM -	8,5	-	3,27	★★	2	0,783	★★	4
CIT_1 2	17,0	★	2,97	★★	3	0,763	★	4
TR_1 1	32,0	★★★	4,53	-	3	0,759	★	4
IT_1 1	12,5	-	3,57	★	1	0,773	★★	3
AF-DM -	19,0	★	4,28	★	2	0,752	★	3
PY3_30 3	10,5	-	1,85	★★★	3	0,734	-	3
TTA_1 2	30,5	★★★	7,53	-	3	0,704	-	3
IT_2 5	20,0	★★	4,40	★	3	0,699	-	3
AF-DM 1	28,0	★★★	5,04	-	3	0,696	-	3
PY_9 3	12,0	-	6,13	-	0	0,781	★★	2
TTA_3 3	26,0	★★	4,81	-	2	0,736	-	2
IT_3 2	10,5	-	4,20	★	1	0,738	-	1
TTA_3 1	23,0	★★	3,35	★★	4			
CIT_1 1	20,5	★★	3,02	★★	4			
IT_1 5	8,0	-	2,15	★★★	3			
PY3_10 2	5,0	-	1,87	★★★	3			
CIT_1 3	18,5	★	3,48	★★	3			
CIT_1 5	18,0	★	3,13	★★	3			
TTA_5 3	15,0	★	2,92	★★	3			
TTA_5 4	15,0	★	2,92	★★	3			
TTA_5 5	13,5	★	2,94	★★	3			
TTA_4 5	23,5	★★	4,39	★	3			
TR_2 2	31,5	★★★	4,80	-	3			
TR_1 3	31,0	★★★	4,92	-	3			
TTA_2 5	30,5	★★★	5,13	-	3			
TR_1 4	30,0	★★★	4,73	-	3			
TTA_1 3	29,5	★★★	6,88	-	3			
TR_1 2	29,0	★★★	4,73	-	3			
TTA_2 4	28,5	★★★	4,84	-	3			
TTA_2 2	27,5	★★★	5,02	-	3			
TR_2 1	27,0	★★★	4,67	-	3			
TTA_2 3	27,0	★★★	4,95	-	3			
IT_1 3	12,0	-	3,37	★★	2			
IT_1 2	8,0	-	3,39	★★	2			

Model	Total stars	V1	Distance between R119 & S230 [Å][1]	V2	V1+V2	AUC	V3	V1+V2+V3
PY3_30 1	5,0	-	3,14	★★	2			
IT_2 2	14,0	★	4,47	★	2			
TR_2 4	26,0	★★	7,53	-	2			
TTA_4 3	26,0	★★	4,69	-	2			
TTA_3 4	24,5	★★	5,16	-	2			
TTA_1 5	24,0	★★	4,71	-	2			
TTA_1 4	23,5	★★	6,86	-	2			
TTA_3 2	23,5	★★	7,28	-	2			
TTA_4 4	21,0	★★	4,63	-	2			
TTA_4 1	20,0	★★	7,04	-	2			
IT_3 3	11,0	-	4,44	★	1			
PY3_30 4	9,5	-	4,23	★	1			
PY3_10 4	6,0	-	4,11	★	1			
PY3_10 3	2,0	-	4,04	★	1			
TTA_3 5	19,5	★	7,57	-	1			
IT_2 4	14,5	★	4,73	-	1			
TTA_5 2	14,0	★	4,81	-	1			
PY_9 1	13,0	★	6,67	-	1			
IT_2 1	12,0	-	5,94	-	0			
IT_2 3	12,0	-	4,73	-	0			
PY_9 4	9,0	-	5,53	-	0			
IT_3 1	8,5	-	10,88	-	0			
PY3_30 5	8,5	-	6,69	-	0			
PY3_30 2	8,0	-	8,56	-	0			
PY_9 2	7,0	-	6,51	-	0			
IT_3 4	6,0	-	9,07	-	0			
IT_3 5	6,0	-	8,82	-	0			
PY_9 5	6,0	-	5,29	-	0			
PY3_10 5	2,0	-	5,05	-	0			

[1] Distance measured between the oxygen atom Ser230 and the nearest hydrogen atom Arg119. The distance between the oxygen atom Ser230 (acceptor) and the nitrogen atom Arg119 (donor) and the angle formed between the three atoms (N, H, O) were also measured, but for the purposes of the ranking system it was decided to award the corresponding number of stars based on only one value.

4. Ligand library generated using publicly available ChEMBL database for enrichment purposes

Table S13 Chemical compounds exhibiting antagonistic or inverse agonistic activity (with experimen-tally determined IC50 < 10μM) classified as active ligands in the enrichment test.

ChEMBL ID	Molecule Name	SMILES	IC_{50} ± SEM [μM]	I_{max} [%]
-	CB-148	O=C(N1C(SCCC1)=N/2)C2=C\C3=CC=CC(OCCCOC4=CC=C(C5=CC=C(C#N)C=C5)C=C4)=C3	0.093 ± 0.029	-
4174825	CB-92	O=C1N2C(SCCC2)=N/C1=C\C3=CC(OCCCCCCOC4=CC=C(C)C=C4)=CC=C3	0.238 ± 0.098	81
3221188	CB-5	O=C1N2CCCSC2=N/C1=C\C3=CC=CC(OCC4=CC=C(Cl)C=C4)=C3	0.279 ± 0.111	60
4168457	CB-27	O=C1N2C(SCCC2)=N/C1=C\C3=CC(OCCCCCCOC4=CC=C(Cl)C=C4)=CC=C3	0.650 ± 0.134	122
4176317	CB-26	O=C1N2C(SCCC2)=N/C1=C\C3=CC(OCCCCCOC4=CC=C(Cl)C=C4)=CC=C3	1.14 ± 0.44	123
4169939	CB-29	O=C1N2C(SCCC2)=N/C1=C\C3=CC(OCCCCCCCCOC4=CC=C(Cl)C=C4)=CC=C3	1.15 ± 0.17	124
4169536	CB-24	O=C1N2C(SCCC2)=N/C1=C\C3=CC(OCCCC4=CC=CC=C4)=CC=C3	1.49 ± 0.39	136
3221189	CB-15	O=C1N2CCCSC2=N/C1=C\C3=CC=CC(OCC4=C(Cl)C=CC=C4)=C3	1.65 ± 0.45	-
4159298	CB-101	O=C1N2C(SCCC2)=N/C1=C\C3=CC(OCCCCCCCOC4=CC=C(Cl)C=C4)=CC=C3	1.71 ± 0.25	97
4168388	CB-20	BrC1=CC=C(COC2=CC=CC(/C=C3N=C(SCCC4)N4C\3=O)=C2)C=C1	1.73 ± 0.37	60
4162436	CB-71	ClC1=CC=C(COC2=CC=CC(/C=C3N=C(SCC4=CC=C(Br)C=C4)NC\3=O)=C2)C=C1	2.29 ± 0.72	44
4171815	CB-28	O=C1N2C(SCCC2)=N/C1=C\C3=CC(OCCCCOC4=CC=C(Cl)C=C4)=CC=C3	2.38 ± 1.15	116
3221190	CB-4	O=C1N2CCCSC2=N/C1=C\C3=CC=CC(OCC4=C(Cl)C=C(Cl)C=C4)=C3	2.55 ± 0.71	-
4161205	CB-25	O=C1N2C(SCCC2)=N/C1=C\C3=CC(OCCCOC4=CC=C(Cl)C=C4)=CC=C3	3.25 ± 1.22	113
4172931	CB-22	CC1=CC=C(COC2=CC=CC(/C=C3N=C(SCCC4)N4C\3=O)=C2)C=C1	3.59 ± 2.25	61
3221201	ChM-73	O=C1N2CCCCSC2=N/C1=C\C3=CC=CC(OCC4=C(Cl)C=C(Cl)C=C4)=C3	4.61 ± 2.36	-
4177155	CB-78	ClC1=CC=C(COC2=CC=CC(/C=C3N=C(SCCC4=CC=CC=C4)NC\3=O)=C2)C=C1	4.78 ± 1.82	103
4161619	CB-30	O=C1N2C(SCCC2)=N/C1=C\C3=CC(OCCOC4=CC=C(Cl)C=C4)=CC=C3	5.00 ± 0.39	123
559612	CP-55940	CCCCCCC(C)(C1=CC(O)=C(C2CC(CCC2CCCO)O)C=C1)C	5.99 ± 1.88	-
3221191	CB-19	O=C1N2CCCSC2=N/C1=C\C3=CC=CC(OCC4=CC(Cl)=C(Cl)C=C4)=C3	6.16 ± 2.86	-
3221192	CB-2	O=C1N2CCCSC2=N/C1=C\C3=CC=CC(OC(C4=CC=CC=C4)=O)=C3	6.35 ± 1.86	-
3220943	-	O=C1N2CCCCSC2=N/C1=C\C3=CC=CC(OC(C4=CC=C(C)C=C4)=O)=C3	6.45 ± 1.10	-
3221200	CB-9	O=C1N2CCCCSC2=N/C1=C\C3=CC=CC(OCC4=C(Cl)C=CC=C4)=C3	6.48 ± 1.20	-
3221187	CB-3	O=C1N2CCCSC2=N/C1=C\C3=CC=CC(OCC4=CC=CC=C4)=C3	7.22 ± 3.42	-
2177264	-	CCCCCCC(C)(C1=CC(O)=C2C=C(C(OC2=C1)=O)CC3=CC=CC=C3)C	8.10 ± 0.58	-
1258979	JBIR-03	C/C(C)=C/C1CC2(C)C(O1)CCC3(C)C2CCC4CC5=C(C34C)NC6=C5C=CC=C6	9.91 ± 2.59	-

Table S14 Chemical compounds not exhibiting antagonistic or inverse agonistic activity (with exper-imentally determined IC50 ≥ 10μM) classified as inactive ligands in the enrichment test.

ChEMBL ID	SMILES	IC_{50} [μM]	I_{max} [%]
2387530	CCCCCC1=CC2=C(C=C(C(O2)=O)CC3=CC=C(Cl)C=C3)C(OC)=C1	≈10	50
3221186	O=C1N2CCCSC2=N/C1=C\C3=CC(OC4=CC=CC=C4)=CC=C3	≈10	54
3221197	O=C1N2CCCCSC2=N/C1=C\C3=CC=CC(OCC4=CC=CC=C4)=C3	≈10	49
4160778	O=C1N2C(SCCC2)=N/C1=C\C3=CC(OCCCCCCOC4=C(C)C=CC=C4C)=CC=C3	≈10	55
111	CC1=C(C2=CC=C(Cl)C=C2)N(C3=C(Cl)C=C(Cl)C=C3)N=C1C(NN4CCCCC4)=O	10.1	-
2387744	COC1=CC=CC=C1CC2=CC3=C(OC2=O)C(C(C)C)=CC=C3C	11.3	-
2181542	CCCCCCCC(C)(C1=CC(O)=C2C=C(C(OC2=C1)=O)CC3=CC=CC=C3O)C	12.5	-
3120632	COC(C)(/C=C/CC1(C(CCC2(C1CCC3CC4=C(C23C)NC5=C4C=CC=C5)C)O)C)C	13.4	-
4163505	ClC1=CC=C(COC2=CC=CC(/C=C3NC(SC)=NC\3=O)=C2)C=C1	13.8	71
3221196	O=C1N2CCCCSC2=N/C1=C\C3=CC=CC(OC4=CC=CC=C4)=C3	14.1	-
3217791	O=C1N2CCCSC2=N/C1=C\C3=CC=CC(OC(C4=CC=C(Cl)C=C4)=O)=C3	14.3	-
2181540	CCCCCCC(C)(C1=CC2=C(C=C(C(O2)=O)CC3=CC=CC=C3O)C(O)=C1)C	15.9	-
4164171	O=C1N2C(SCCC2)=N/C1=C\C3=CC(OCCCCCCOC4=CC=C(CC)C=C4)=CC=C3	25.0	99
4159707	ClC1=CC=C(COC2=CC=CC(/C=C3NC(NC\3=O)=S)=C2)C=C1	33.0	165
2387534	COC1=C(C(C)=C2OC(C(CC3=CC=CC=C3)=CC2=C1C)=O)C	⩾10	46
2387537	CC(C(C)=C1O2)=C(O)C(C)=C1C=C(CC3=CC=C(C=C3)F)C2=O	⩾10	47
2181544	CCCCC1(C2=CC(O)=C3C=C(C(OC3=C2)=O)CC4=C(O)C=CC=C4)CCCC1	⩾10	57
2181546	CCCCC1(C2=CC(O)=C3C=C(C(OC3=C2)=O)CC4=CC=CC=C4O)CCCCC1	⩾10	59
4170995	O=C1N2C(SCCC2)=N/C1=C\C3=CC(OCCCCCCOC4=CC=CC(Cl)=C4)=CC=C3	>10	55
4163080	O=C1N2C(SCCC2)=N/C1=C\C3=CC(OCCCCCCOC4=C(Cl)C=CC=C4)=CC=C3	>10	41
4176334	ClC1=CC=C(COC2=CC=CC(/C=C3N=C(SCC4)N4C\3=O)=C2)C=C1	>10	40
2387539	CC1=C(CC2=CC3=C(OC2=O)C(C)=C(C)C(O)=C3C)C=CC=C1	>10	38
1257246	C/C(C)=C\CCC1(C(CCC2(C1CCC3CC4=C(C23C)NC5=C4C=CC=C5)C)O)C	>10	37
3221195	O=C1N2CCCCSC2=N/C1=C\C3=CC=C(C4=CC=CC=C4)C=C3	>10	37
4170355	ClC1=CC=C(COC2=CC=CC(/C=C3N=C(SCC4=CC=C(Cl)C=C4)NC\3=O)=C2)C=C1	>10	35
2177253	CCCCCC1=CC(OC)=C2C=C(C(OC2=C1)=O)CC3=CC=CC=C3C	>10	34
2387538	COC1=C(C(C)=C2OC(C(CC3=C(C)C=CC=C3)=CC2=C1C)=O)C	>10	33
2177249	CCCCCC1=CC(OC)=C2C=C(C(OC2=C1)=O)CC3=CC=CC(OC)=C3	>10	33
2181541	CCCCCCCC(C)(C1=CC2=C(C=C(C(O2)=O)CC3=CC=CC=C3OC)C(OC)=C1)C	>10	33
2181545	CCCCC1(C2=CC(OC)=C3C=C(C(OC3=C2)=O)CC4=C(OC)C=CC=C4)CCCCC1	>10	33
2181549	CC(C(C=CC(C)=C1O2)=C1C=C(CC3=CC=CC=C3O)C2=O)C	>10	32
2387536	COC1=C(C(C)=C2OC(C(CC3=CC=C(F)C=C3)=CC2=C1C)=O)C	>10	32
2181551	COC1=CC(CC2=CC3=C(OC2=O)C=C(Br)C=C3OC)=CC=C1	>10	31
2177263	CCCCCCC(C)(C1=CC(OC)=C2C=C(C(OC2=C1)=O)CC3=CC=CC=C3)C	>10	31
3221198	O=C1N2CCCCSC2=N/C1=C\C3=CC=CC(OCC4=CC=C(Cl)C=C4)=C3	>10	31
2387532	CCCCCC1=CC(OC)=C2C=C(C(OC2=C1)=O)CC3=CC=C(C=C3)Br	>10	30
2387533	COC1=CC=CC=C1CC2=CC3=C(OC2=O)C(C)=C(C)C(OC)=C3C	>10	27
2177250	CCCCCC1=CC(O)=C2C=C(C(OC2=C1)=O)CC3=CC=CC(O)=C3	>10	27
4159050	ClC1=CC=C(COC2=CC=CC(/C=C3N=C(SCC4=CC=C(F)C=C4)NC\3=O)=C2)C=C1	>10	27
2181539	CCCCCCC(C)(C1=CC2=C(C=C(C(O2)=O)CC3=CC=CC=C3OC)C(OC)=C1)C	>10	26
2177259	CCCCCC1=CC(OC)=C2C=C(C(OC2=C1)=O)CC3=CC=CC=C3Cl	>10	25
2181543	CCCCC1(C2=CC(OC)=C3C=C(C(OC3=C2)=O)CC4=CC=CC=C4OC)CCCC1	>10	25
4169314	ClC1=CC=C(COC2=CC=CC(/C=C3N(CCS4)C4=NC\3=O)=C2)C=C1	>10	25
470199	COC1=CC(C)=CC(O2)=C1C=C(CC3=CC=CC=C3)C2=O	>10	24
4171382	O=C1N2C(SCCC2)=N/C1=C\C3=CC(OCCCCCCOC4=CC=C(C(C)C)C=C4)=CC=C3	>10	24

ChEMBL ID	SMILES	IC_{50} [μM]	I_{max} [%]
4174205	ClC1=CC=C(COC2=CC=CC(/C=C3N=C(SC)N(C)C\3=O)=C2)C=C1	>10	23
3221203	O=C1N2CCCCSC2=N/C1=C\C3=CC=CC(OCC(OCC)=O)=C3	>10	22
4173561	ClC1=CC=C(COC2=CC=CC(/C=C3SC(NC\3=O)=S)=C2)C=C1	>10	22
2177257	CCCCCC1=CC(OC)=C2C=C(C(OC2=C1)=O)CC3=CC=CC=C3OC	>10	20
3220957	O=C1N2CCCSC2=N/C1=C\C3=CC=C([N+]([O-])=O)C=C3	>10	20
3221206	O=C1N2CCCSC2=N/C1=C\C3=CC=C(Cl)C=C3	>10	18
3220953	O=C1N2CCCSC2=N/C1=C\C3=C(F)C=CC=C3	>10	17
4165882	ClC1=CC=C(COC2=CC=CC(/C=C3N=C(SCC4=CC=C(C)C=C4)N(C)C\3=O)=C2)C=C1	>10	17
2387535	CC(C(C)=C1O2)=C(O)C(C)=C1C=C(CC3=CC=CC=C3)C2=O	>10	16
4165036	O=C1N2C(SCCC2)=N/C1=C\C3=CC(OCCC4=CC=CC=C4)=CC=C3	>10	16
3120631	C/C(C)=C\CCC1(C(OC2OC(C(C(C2O)O)O)CO)CCC3(C1CCC4CC5=C(C34C)NC6=C5C=CC=C6)C)C	>10	15
3221199	O=C1N2CCCCSC2=N/C1=C\C3=CC=CC(OCC4=CC(Cl)=CC=C4)=C3	>10	15
3221204	CCCCC/C=C\C/C=C\C/C=C\C/C=C\CCCC(OC(COP(O)(OC1C(C(C(C(C1O)O)O)O)O)=O)CO)=O	>10	15
3221207	O=C1N2CCCSC2=N/C1=C\C3=CC(Cl)=CC=C3	>10	14
3221210	O=C1N2CCCSC2=N/C1=C\C3=C(Cl)C=C(Cl)C=C3	>10	14
3220956	O=C1N2CCCSC2=N/C1=C\C3=CC=C(Br)C=C3	>10	13
3221185	O=C1N2CCCSC2=N/C1=C\C3=CC=C(N(C)C)C=C3	>10	13
470224	CC(C(C=CC(C)=C1O2)=C1C=C(CC3=CC=CC=C3)C2=O)C	>10	12
3220947	O=C1N2C(SCC2)=NC(C3=CC=CC=C3)1C4=CC=CC=C4	>10	12
506746	CC(C(C=CC(C)=C1O2)=C1C=C(C)C2=O)C	>10	11
2177254	CCCCCC1=CC2=C(C=C(C(O2)=O)CC3=CC=CC=C3C)C(O)=C1	>10	10
3220959	O=C1N2CCCSC2=N/C1=C\C3=CC=C(OC)C=C3	>10	10
3221182	O=C1N2CCCSC2=N/C1=C\C3=C(OC)C(OC)=CC=C3	>10	10
3221183	O=C1N2CCCSC2=N/C1=C\C3=C(OC)C=C(OC)C=C3	>10	7
3221208	O=C1N2CCCSC2=N/C1=C\C3=C(Cl)C=CC=C3	>10	6
3220955	O=C1N2CCCSC2=N/C1=C\C3=C(F)C=C(F)C=C3	>10	6
3220958	O=C1N2CCCSC2=N/C1=C\C3=CC([N+]([O-])=O)=CC=C3	>10	6
3221205	O=C1N2CCCSC2=N/C1=C\C3=CC=CC=C3	>10	5
3221202	O=C1N2CCCCSC2=N/C1=C\C3=CC=CC(OCC4=CC=C(F)C=C4)=C3	>10	5
3221184	O=C1N2CCCSC2=N/C1=C\C3=CC=C(C(C)C)C=C3	>10	4
3220944	O=C1N2CCCCSC2=N/C1=C(C)\C3=CC=CC=C3	>10	4
4169940	ClC1=CC=C(COC2=CC=CC(/C=C3N(C)C(NC\3=O)=N)=C2)C=C1	>10	4
3220954	O=C1N2CCCSC2=N/C1=C\C3=C(F)C(F)=CC=C3	>10	3
4160494	FC1=CC=C(COC2=CC=CC(/C=C3N=C(SCCC4)N4C\3=O)=C2)C=C1	>10	3
4166952	ClC1=CC=C(COC2=CC=CC(/C=C3N=C(SCC4=CC=CC=C4)NC\3=O)=C2)C=C1	>10	3
3221181	O=C1N2CCCSC2=N/C1=C\C3=CC(OC)=C(OC)C=C3	>10	2
3221194	O=C1N=C2SCCCN2/C1=C\C=C\C3=CC=CC=C3	>10	2
161343	CCCCC/C=C\C/C=C\C/C=C\C/C=C\CCCC(NCC(O)=O)=O	>10	0
2387743	COC1=C(CC2=CC3=C(C=CC(C)=C3OC2=O)C)C=CC=C1	>10	0
2177260	CCCCCC1=CC2=C(C=C(C(O2)=O)CC3=C(Cl)C=CC=C3)C(O)=C1	>10	0
3221209	O=C1N2CCCSC2=N/C1=C\C3=C(Cl)C(Cl)=CC=C3	>10	0
3221211	O=C1N2CCCSC2=N/C1=C\C3=C(Cl)C=CC=C3Cl	>10	0
3221180	O=C1N2CCCSC2=N/C1=C\C3=C(OC)C=CC=C3	>10	0
3221193	O=C1N2CCCSC2=N/C1=C\C=C\C3=CC=CC=C3	>10	0

ChEMBL ID	SMILES	IC_{50} [μM]	I_{max} [%]
3220945	O=C1N=C2N(CCS2)C(C3=CC=CC=C3)1C4=CC=CC=C4	>10	0
3220946	O=C1N=C2N(CC(C(OCC)=O)S2)C(C3=CC=CC=C3)1C4=CC=CC=C4	>10	0
3220948	O=C1N2C(SC(C(OCC)=O)C2)=NC(C3=CC=CC=C3)1C4=CC=CC=C4	>10	0
3220949	O=C1N=C2N(CCCS2)C(C3=CC=CC=C3)1C4=CC=CC=C4	>10	0
3220950	O=C1N2C(SCCC2)=NC(C3=CC=CC=C3)1C4=CC=CC=C4	>10	0
3220951	O=C1N=C2N(CCCCS2)C(C3=CC=CC=C3)1C4=CC=CC=C4	>10	0
3220952	O=C1N2C(SCCCC2)=NC(C3=CC=CC=C3)1C4=CC=CC=C4	>10	0
4173804	ClC1=CC=C(COC2=CC=CC(/C=C3N=C(SCC4=CC=C(C)C=C4)NC\3=O)=C2)C=C1	>10	0
4159299	ClC1=CC=C(COC2=CC=CC(/C=C3SC(NC\3=O)=O)=C2)C=C1	>10	-10

5. ROC plots and AUC values for 8 selected models

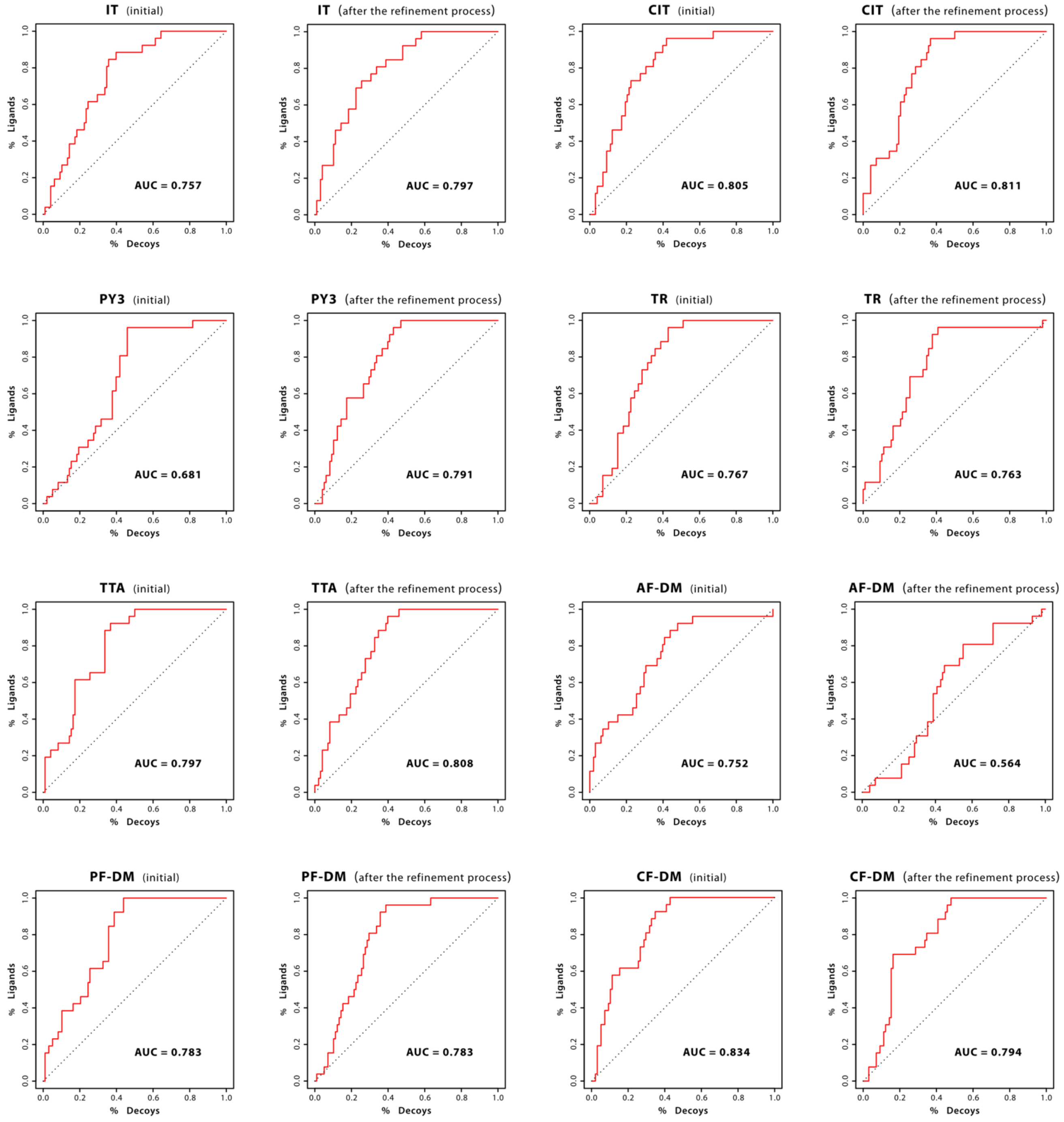

Figure S6 ROC plots from enrichment tests performed before and after the refinement process for 8 selected models, along with calculated AUC values.

6. Visual representation of the selected models

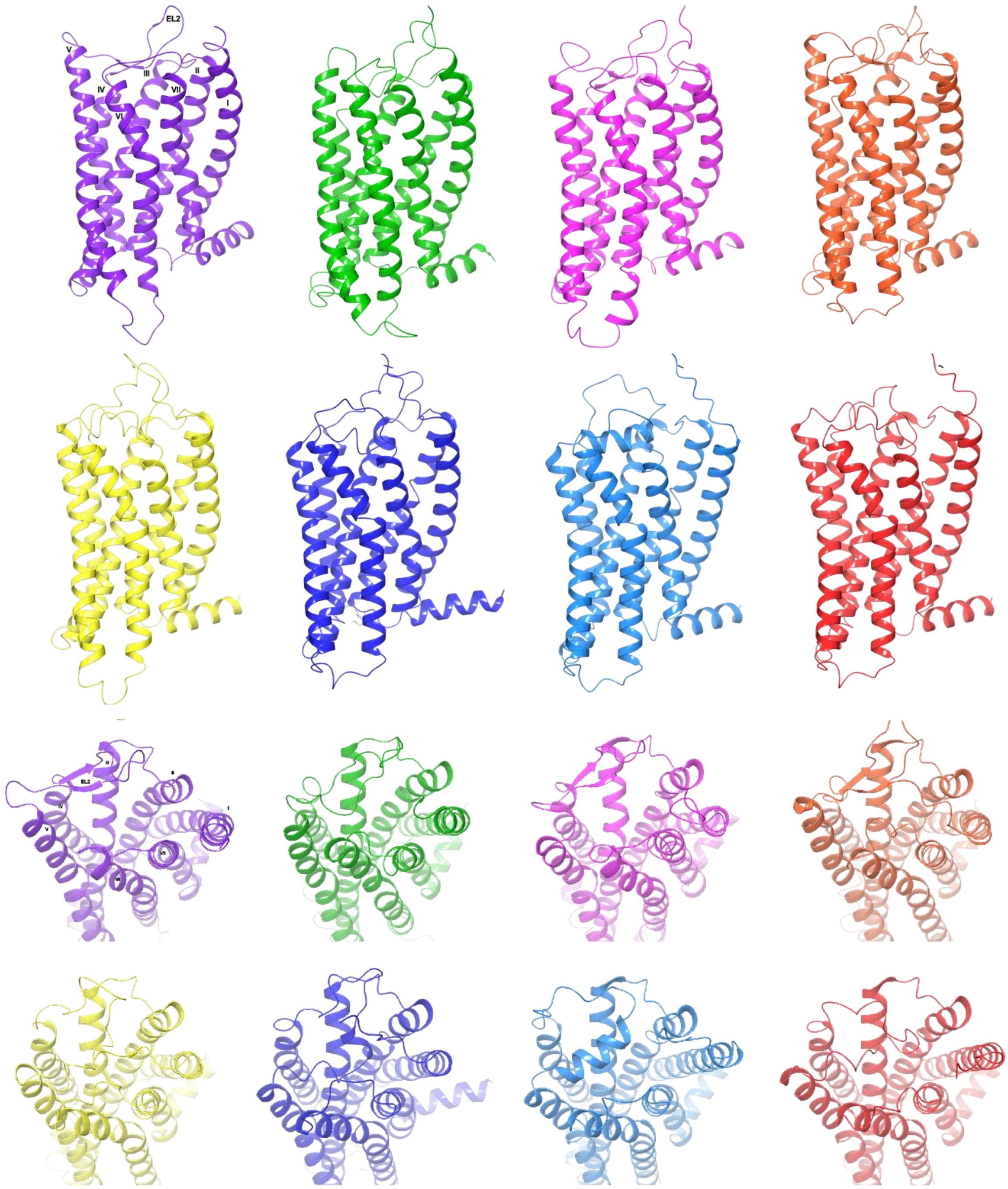

Figure S7 Lateral projection of described models. L to R: (top row) IT, CIT, PY3, TR, (bottom row) TTA, AF-DM, PF-DM, CF-DM. Roman numerals indicate TMs - only marked in IT model for clarity purposes. For colored figure please refer to online version of this paper

7. GPCR Lipid family structures used for comparative purposes

Table S15 List of GPCR lipid family structures with basic information on method, resolution, state and bound ligand along with PDB ID's and IUPHAR classification

PDB ID	IUPHAR	Receptor family	Method	Resolution [Å]	State	Degree active [%]	Structure ligand (function)
**6KQI**	CB1	Cannabinoid	X-ray	3.3	Inactive	14	Agonist. NAM
**5U09**	CB1	Cannabinoid	X-ray	2.6	Inactive	14	Inverse agonist
**5TGZ**	CB1	Cannabinoid	X-ray	2.8	Inactive	14	Antagonist
**5XR8**	CB1	Cannabinoid	X-ray	3.0	Active	96	Agonist
**5XRA**	CB1	Cannabinoid	X-ray	2.8	Active	96	Agonist
**6KPG**	CB1	Cannabinoid	cryo-EM	3.0	Active	100	Agonist
**6N4B**	CB1	Cannabinoid	cryo-EM	3.0	Active	100	Agonist
**6KPC**	CB2	Cannabinoid	X-ray	3.2	Inactive	5	Agonist
**5ZTY**	CB2	Cannabinoid	X-ray	2.8	Inactive	5	Antagonist
**6KPF**	CB2	Cannabinoid	cryo-EM	2.9	Active	100	Agonist
**6PT0**	CB2	Cannabinoid	cryo-EM	3.2	Active	100	Agonist
**4PHU**	FFA1	Free fatty acid	X-ray	2.3	Intermediate	32	Agonist
**5TZR**	FFA1	Free fatty acid	X-ray	2.2	Intermediate	32	Agonist
**5TZY**	FFA1	Free fatty acid	X-ray	3.2	Intermediate	32	Agonist. PAM
**5KW2**	FFA1	Free fatty acid	X-ray	2.8	Intermediate	32	Allosteric agonist
**6RZ4**	CysLT1	Leukotriene	X-ray	2.7	Intermediate	32	Antagonist
**6RZ5**	CysLT1	Leukotriene	X-ray	2.5	Intermediate	32	Antagonist
**6RZ6**	CysLT2	Leukotriene	X-ray	2.4	Intermediate	32	Antagonist
**6RZ7**	CysLT2	Leukotriene	X-ray	2.4	Intermediate	32	Antagonist
**6RZ8**	CysLT2	Leukotriene	X-ray	2.7	Intermediate	32	Antagonist
**6RZ9**	CysLT2	Leukotriene	X-ray	2.7	Intermediate	32	Antagonist
**7K15**	BLT1	Leukotriene	X-ray	2.9	Inactive	6	Antagonist
**4Z34**	LPA1	Lysophospholipid	X-ray	3.0	Inactive	1	Antagonist
**4Z35**	LPA1	Lysophospholipid	X-ray	2.9	Inactive	1	Antagonist
**4Z36**	LPA1	Lysophospholipid	X-ray	2.9	Inactive	1	Antagonist
**3V2W**	S1P1	Lysophospholipid	X-ray	3.4	Inactive	1	Antagonist
**3V2Y**	S1P1	Lysophospholipid	X-ray	2.8	Inactive	1	Antagonist
**7C4S**	S1P3	Lysophospholipid	X-ray	3.2	Active	81	Agonist
**7EW2**	S1P3	Lysophospholipid	cryo-EM	3.1	Active	100	Agonist
**7EW4**	S1P3	Lysophospholipid	cryo-EM	3.2	Active	100	Agonist
**7EW3**	S1P3	Lysophospholipid	cryo-EM	3.1	Active	100	Agonist
**7EW1**	S1P5	Lysophospholipid	cryo-EM	3.4	Active	100	Agonist
**5ZKQ**	PAF	Platelet-activating factor	X-ray	2.9	Intermediate	32	Inverse agonist

**5ZKP**	PAF	Platelet-activating factor	X-ray	2.8	Other	-	Antagonist
**6D26**	DP2	Prostanoid	X-ray	2.8	Inactive	24	Antagonist
**7M8W**	DP2	Prostanoid	X-ray	2.6	Inactive	24	Antagonist
**6D27**	DP2	Prostanoid	X-ray	2.7	Inactive	24	Antagonist
**7CX2**	EP2	Prostanoid	cryo-EM	2.8	Active	100	Agonist
**7CX3**	EP2	Prostanoid	cryo-EM	2.8	Active	100	Agonist
**7CX4**	EP2	Prostanoid	cryo-EM	2.9	Active	100	Agonist
**6M9T**	EP3	Prostanoid	X-ray	2.5	Active	83	Agonist
**6AK3**	EP3	Prostanoid	X-ray	2.9	Active	83	Agonist
**5YHL**	EP4	Prostanoid	X-ray	4.2	Inactive	12	Antagonist
**5YWY**	EP4	Prostanoid	X-ray	3.2	Inactive	12	Antagonist
**7D7M**	EP4	Prostanoid	cryo-EM	3.3	Active	100	Agonist
**6IIV**	TP	Prostanoid	X-ray	3.0	Intermediate	32	Antagonist
**6IIU**	TP	Prostanoid	X-ray	2.5	Intermediate	32	Antagonist

8. Sodium binding site analysis

As, unlike other GPCRs, GPR18 has a DVXXY (D7.49-Y7.53) instead of the NPXXY motif in TM7, the sodium binding region is located lower than in other GPCR's. This is due to the fact, that instead of neutral N7.49, GPR18 has negatively charged D7.49. Therefore, the sodium binding pocket for GPR18 has been proposed between two aspartates: D68$^{2.50}$ and D282$^{7.49}$. Actually, during the MD simulations the sodium ion was stable, with mild shift towards D282$^{7.49}$ at the end of production stages due to delicate rotation of D68$^{2.50}$. Exemplary behavior of sodium atom in 0 and 10 ns of simulation has been presented on Figure S8. Moreover, the rotamer of D282$^{7.49}$ and its distance to Q240$^{6.43}$ allowed for maintaining possible additional intra-helical H-bond between these two residues in case of the "folded" (FM method) models, and surprisingly PY3 model during the simu-lation, as shown on Figure S9 (for the apo models). Interactive graphs showing the distance of sodium atom to two neighboring residues: N40$^{1.50}$ and A108$^{3.39}$ for all models can be found in an attached XLS file.

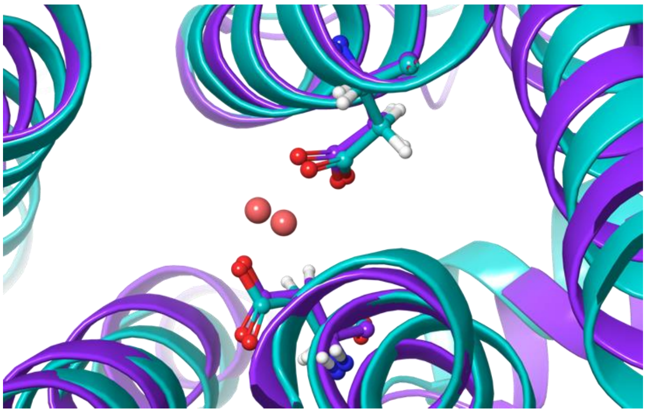

Figure S8 Position of sodium atom (red spheres) in the IT model at the beginning (0ns, violet protein, left sphere) and after 10ns of simulation (teal protein, right sphere). For colored figures please refer to online version of this paper.

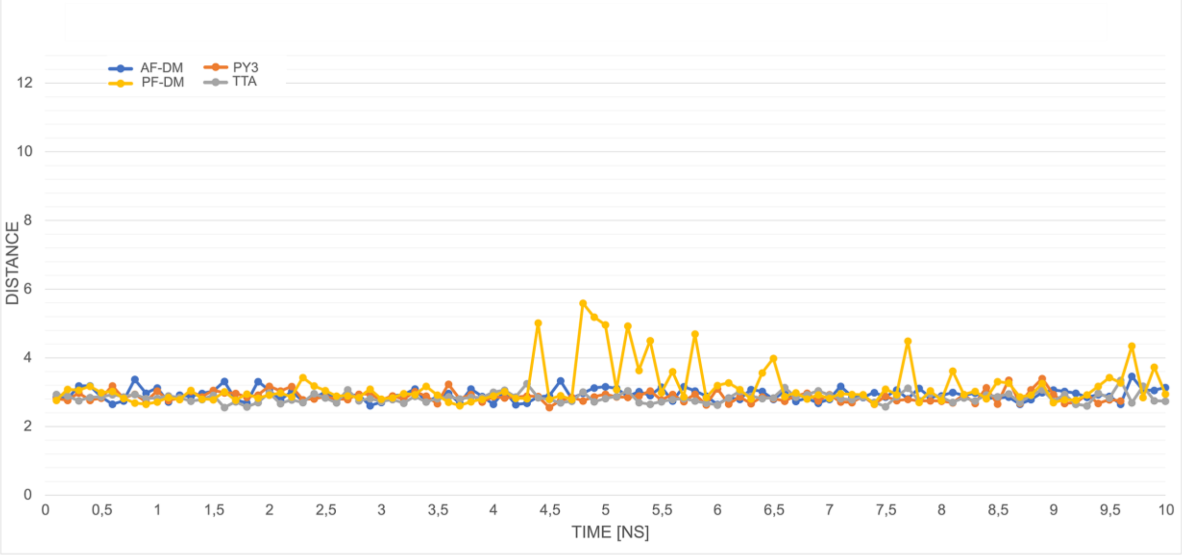

Figure S9 Changes in the D282$^{7.49}$ - Q240$^{6.43}$ distance for AF-DM, PF-DM, TTA and PY3 models during 10ns MD simulation of the apo forms. For colored graph please refer to online version of this paper.

9. Comparison of binding modes

Table S16 Comparison on putative dinging modes for all models/ligands calculated by Dock Thor (green) and GWO Vina (orange)

IT-CB5	IT-CB27	IT-CB92
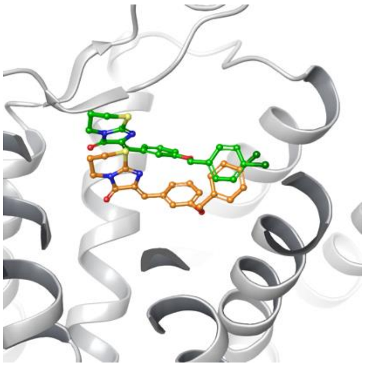	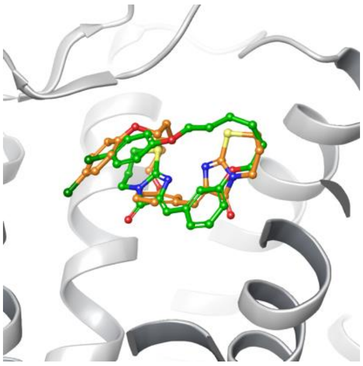	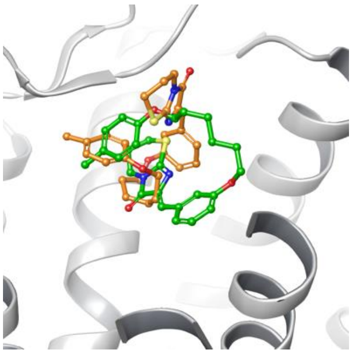
IT-CB148	IT-CB153	IT-CB154
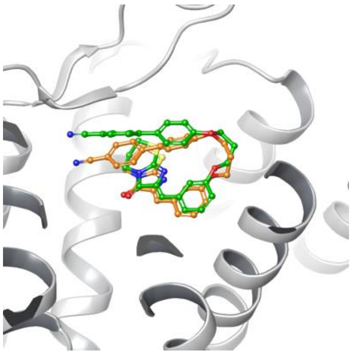	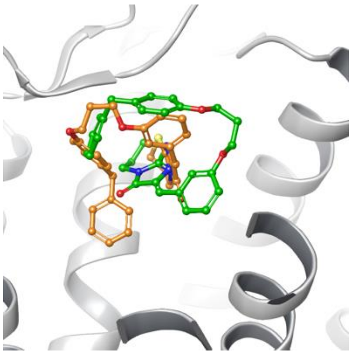	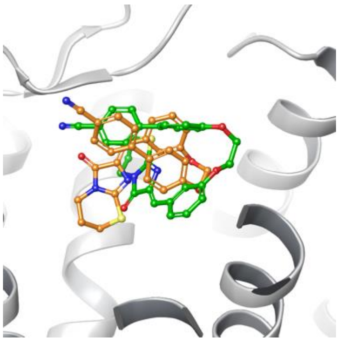

CIT-CB5<br>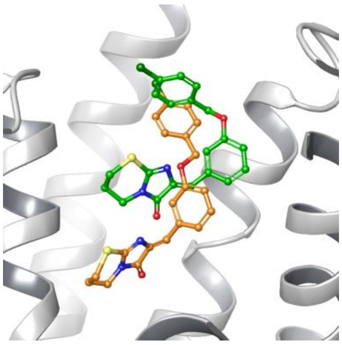	CIT-CB27<br>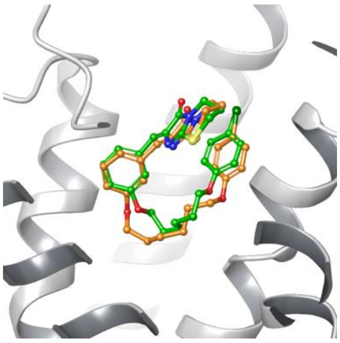	CIT-CB92<br>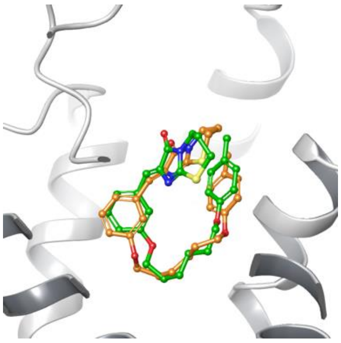
CIT-CB148<br>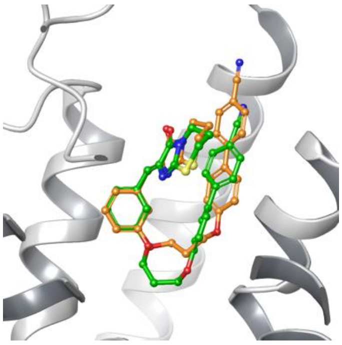	CIT-CB153<br>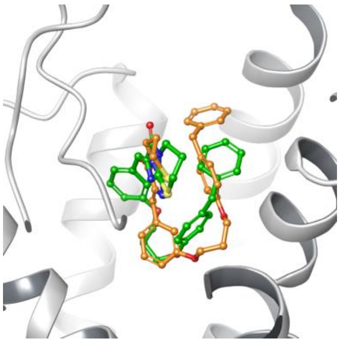	CIT-CB154<br>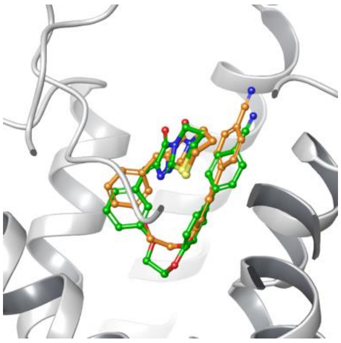
PY3-CB5<br>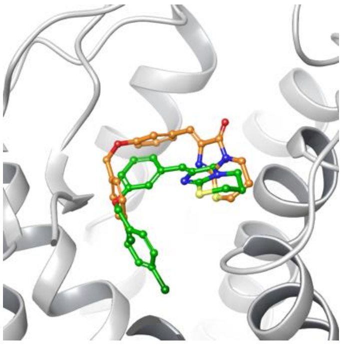	PY3-CB27<br>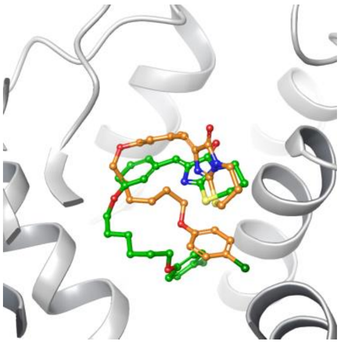	PY3-CB92<br>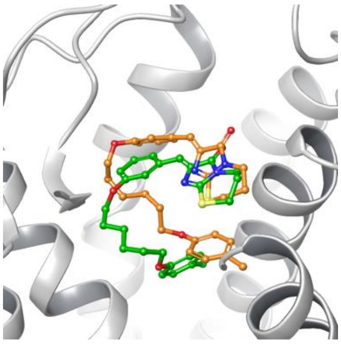
PY3-CB148<br>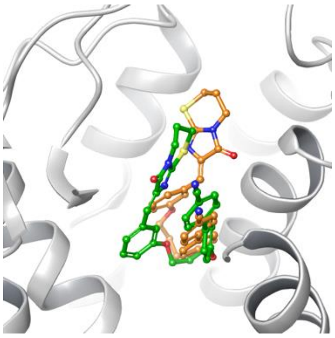	PY3-CB153<br>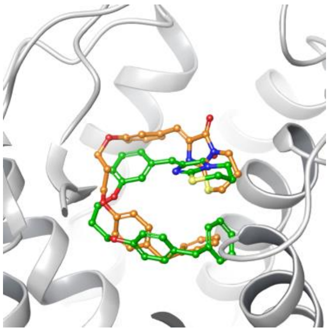	PY3-CB154<br>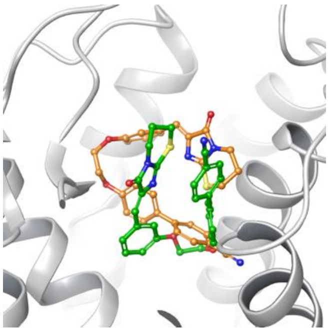

TR-CB5<br>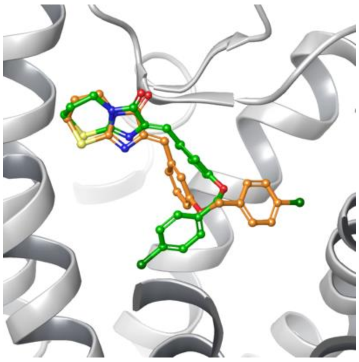	TR-CB27<br>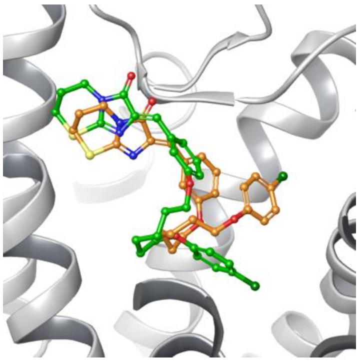	TR-CB92<br>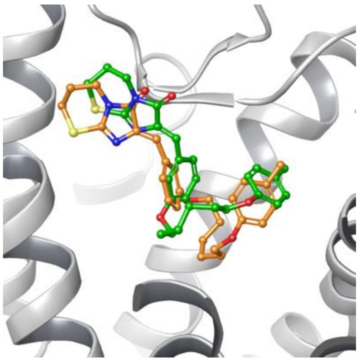
TR-CB148<br>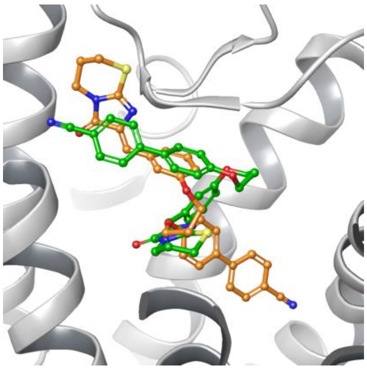	TR-CB153<br>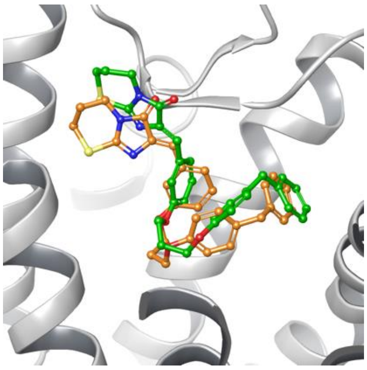	TR-CB154<br>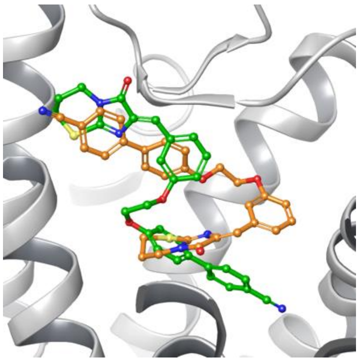
TTA-CB5<br>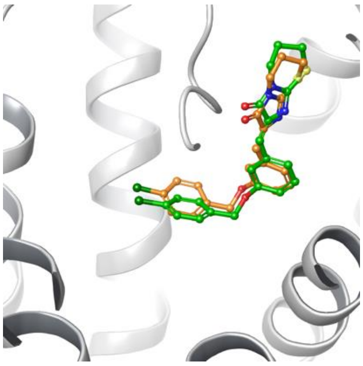	TTA-CB27<br>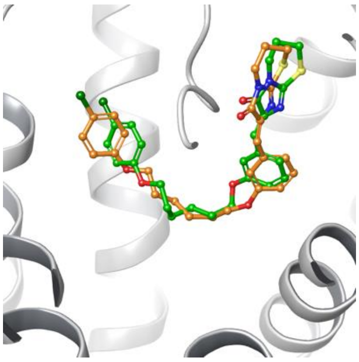	TTA-CB92<br>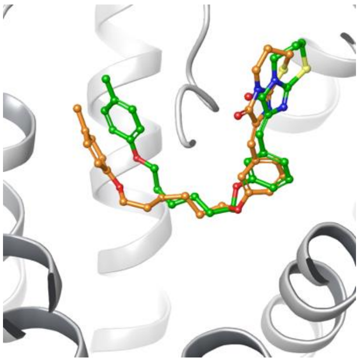
TTA-CB148<br>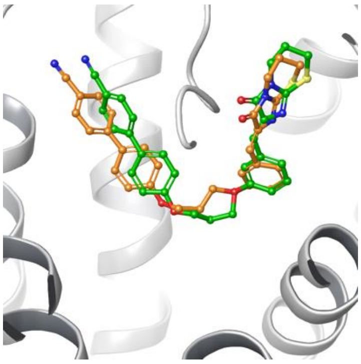	TTA-CB153<br>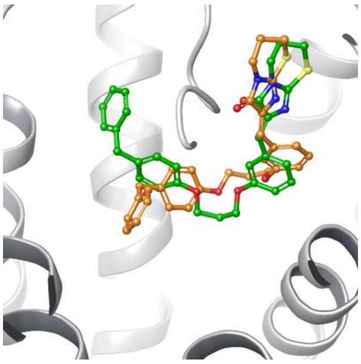	TTA-CB154<br>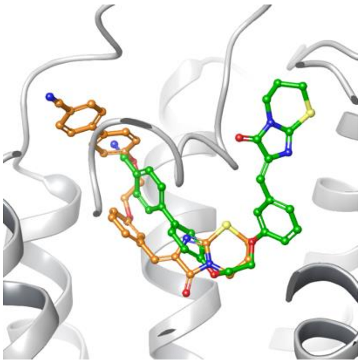

AF-DM-CB5 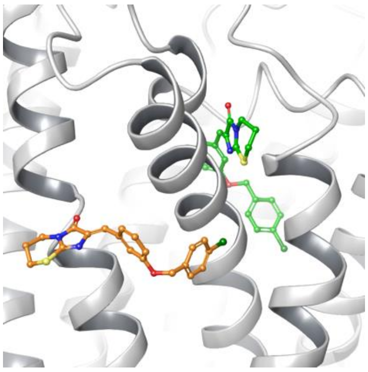	AF-DM-CB27 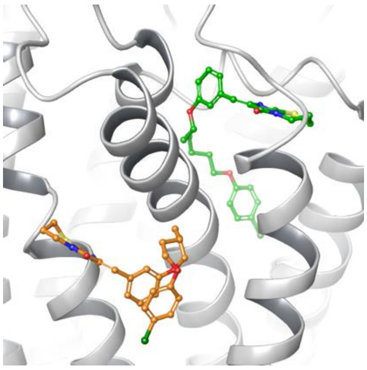	AF-DM-CB92 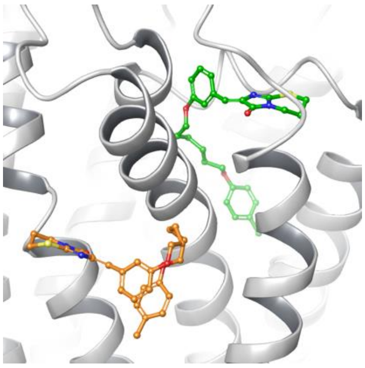
AF-DM-CB148 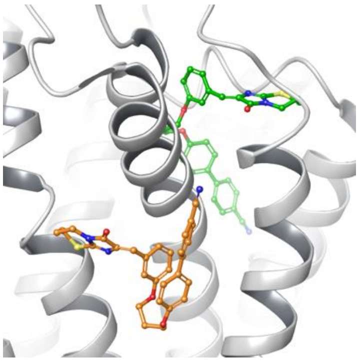	AF-DM-CB153 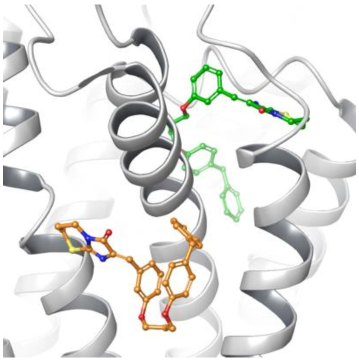	AF-DM-CB154 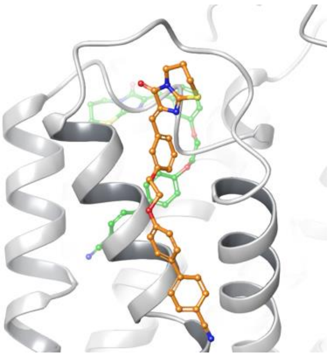
PF-DM-CB5 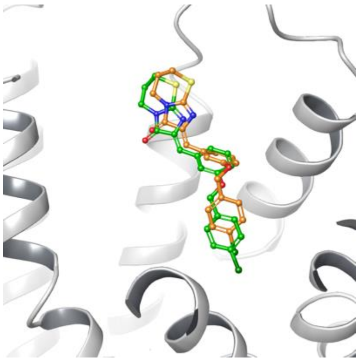	PF-DM-CB27 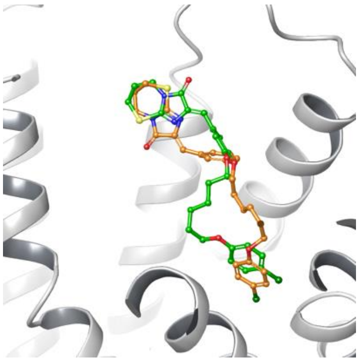	PF-DM-CB92 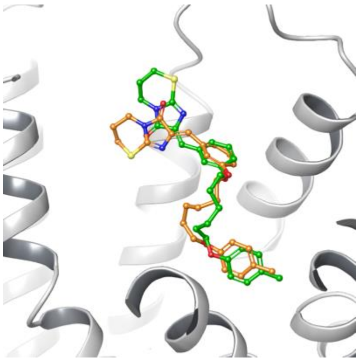
PF-DM-CB148 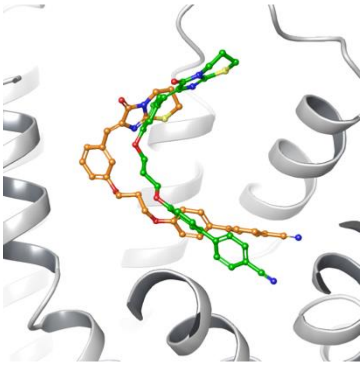	PF-DM-CB153 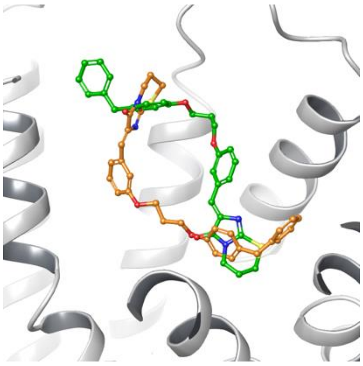	PF-DM-CB154 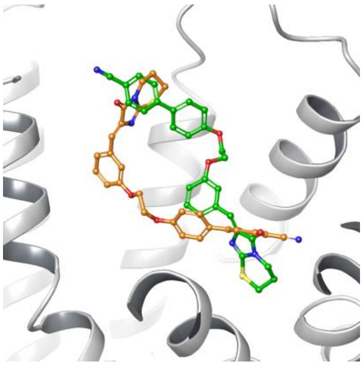

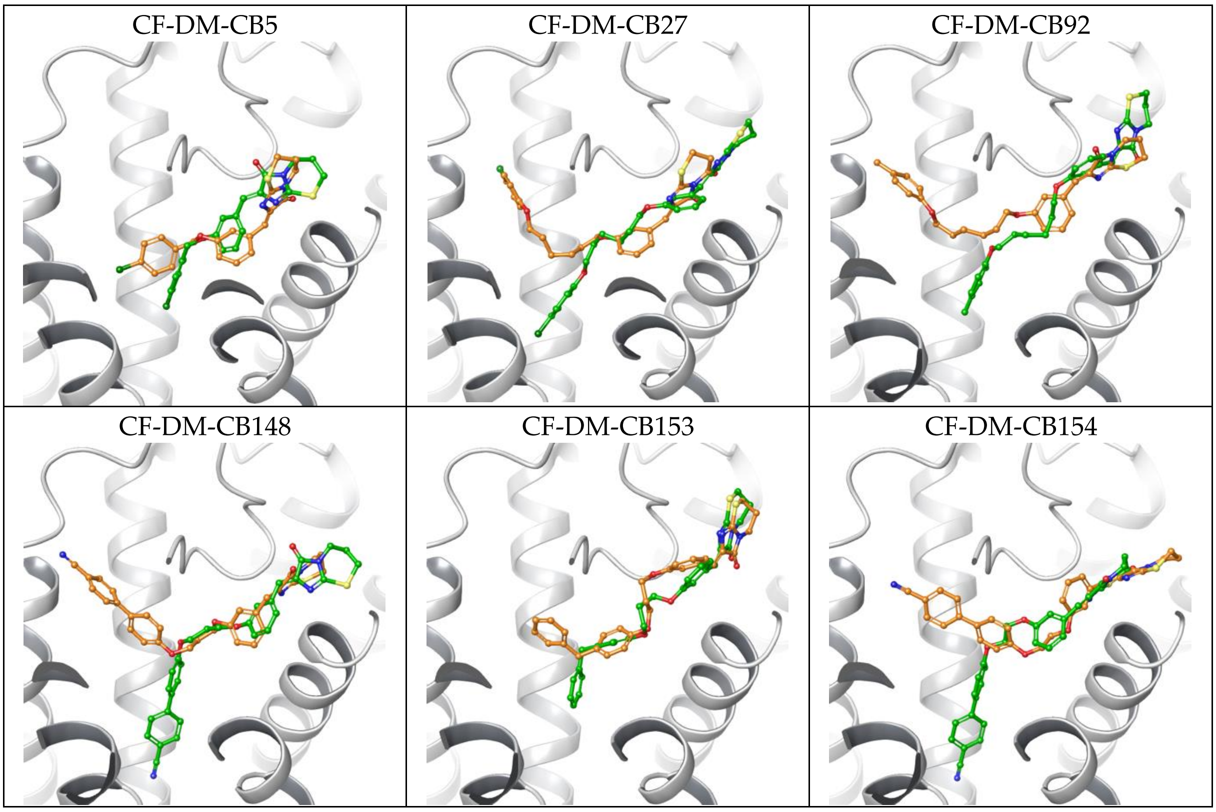
CF-DM-CB5
CF-DM-CB27
CF-DM-CB92
CF-DM-CB148
CF-DM-CB153
CF-DM-CB154

10. Alignment of 0ns-10ns frames for all of the simulated complexes

Table S17 Examination of ligands’ stability in the binding pockets of described models. Compound poses were captured at following frames: 0ns – green, 1ns – teal, 2ns – blue, 3ns – violet, 4ns – magenta, 5ns – pink, 6ns – red, 7ns - orange, 8ns - light orange, 9ns – yellow, 10ns – lime.

IT-CB5<br>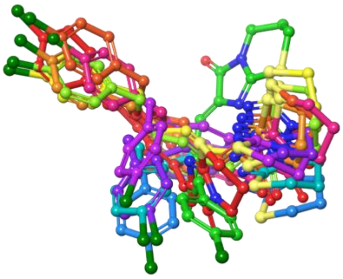	IT-CB148<br>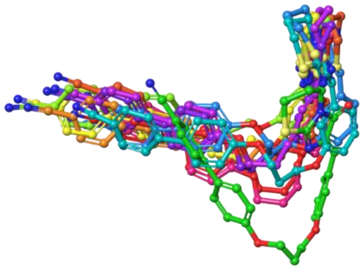	CIT-CB5<br>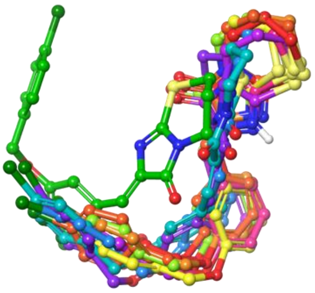
CIT-CB148<br>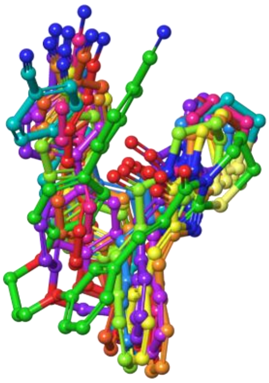	PY3-CB5<br>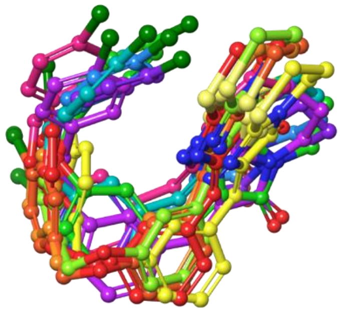	PY3-CB148<br>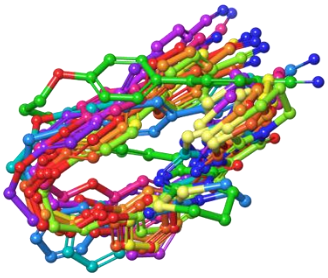
TR-CB5<br>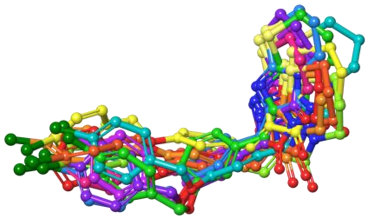	TR-CB148<br>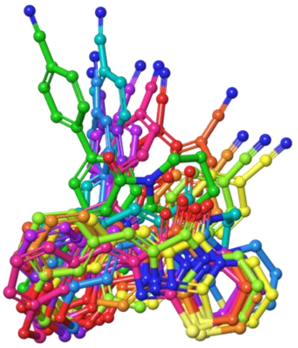	TTA-CB5<br>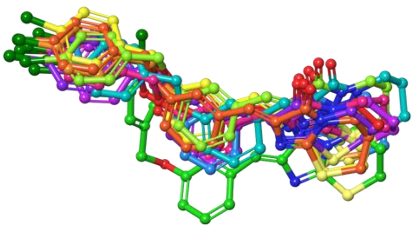

TTA-CB148	AF-DM-CB5	AF-DM-CB148
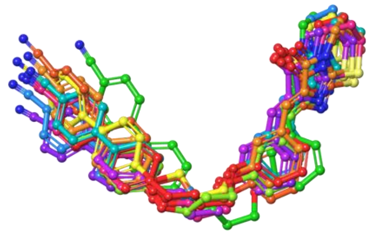	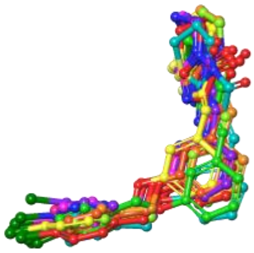	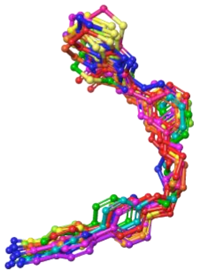
PF-DM-CB5	PF-DM-CB27	PF-DM-CB92
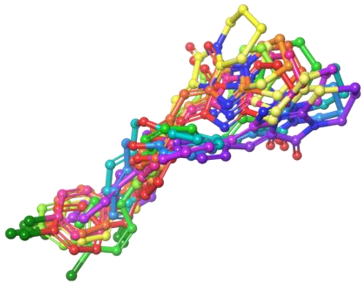	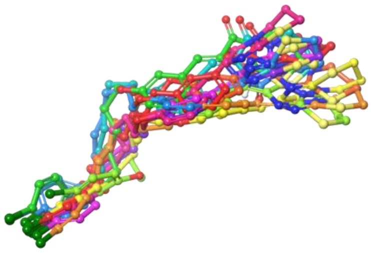	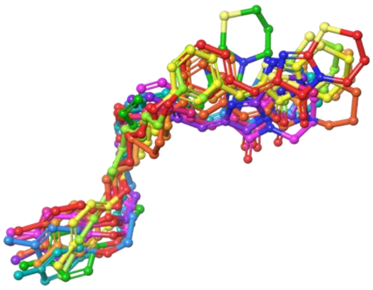
PF-DM-CB148	PF-DM-CB153	PF-DM-CB153 – “reversed”
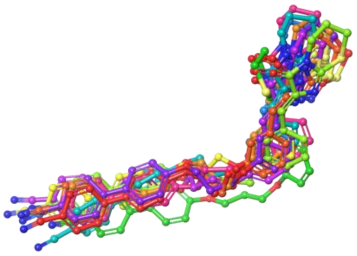	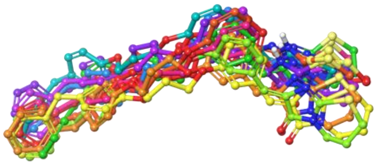	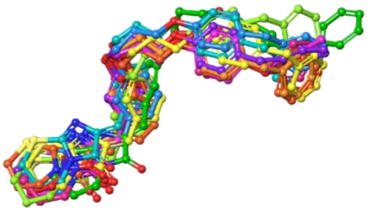
PF_DM-CB154	CF-DM-CB5	CF-DM-CB27
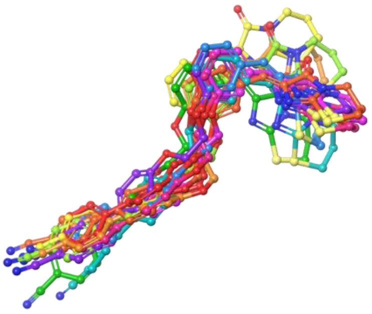	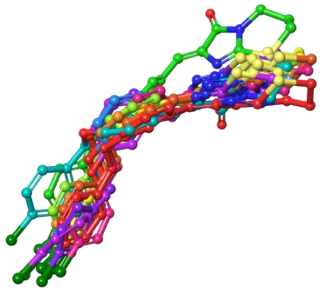	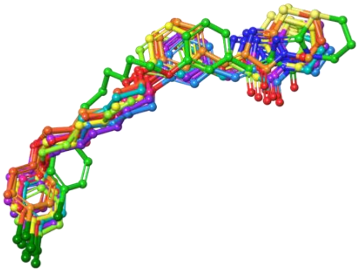
CF-DM-CB92	CF-DM-CB148	CF-DM-CB153
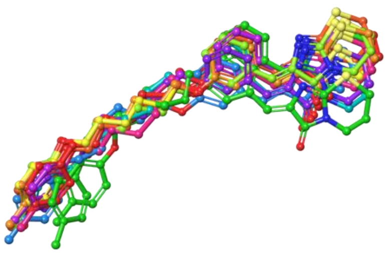	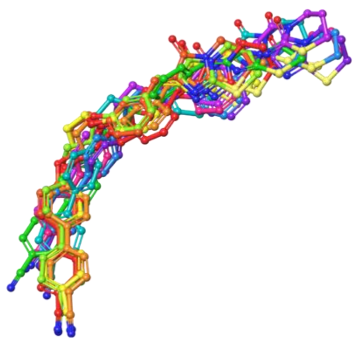	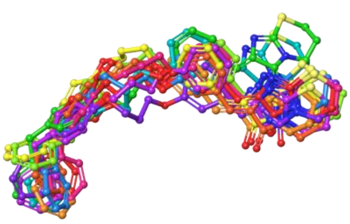

CF-DM-CB154

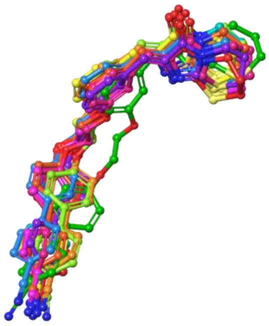

Supplement: Supplementary file 1 [file ijms-23-07917-s001.zip › ijms-1779275-supplementary.pdf]
